# Supplementary material for: Ammonium Catecholaldehydes as Multifunctional Bioactive Agents: Evaluating Antimicrobial, Antioxidant, and Antiplatelet Activity
Source: Int J Mol Sci. 2025 Aug 14;26(16):7866. doi: 10.3390/ijms26167866 (PMC12386629; doi:10.3390/ijms26167866)
Supplement: Supplementary file 1 [file ijms-26-07866-s001.zip › ijms-3755129-supplementary.pdf]

SUPPLEMENTARY MATERIALS

Ammonium Catecholaldehydes as Multifunctional Bioactive Agents: Evaluating Antimicrobial, Antioxidant, and Antiplatelet Activity

Andrei V. Bogdanov [1,*,†], Roza G. Tagasheva [2], Alexandra Voloshina [3], Anna Lyubina [3], Olga Tsivileva [4], Artem N. Kuzovlev [5], Wang Yi [6], Aleksandr V. Samorodov [7,*,†], Guzel K. Ziyatdinova [1], Elnara R. Zhiganshina [8], Maxim V. Arsenyev [8] and Sergey V. Bukharov [2]

[1] Department of High Molecular and Organoelement Compounds, Kazan Federal University, Kremlevskaya Str. 18, Kazan 420008, Russia; Analytical Chemistry Department; guzel.ziyatdinova@kpfu.ru

[2] Department of Technology of Basic Organic and Petrochemical Synthesis, Kazan National Research
Technological University, K. Marx Str. 68, Kazan 420015, Russia;
roza-ta1982@yandex.ru (R.G.T.);
svbukharov@mail.ru (S.V.B.)

[3] Arbuzov Institute of Organic and Physical Chemistry, FRC Kazan Scientific Center, Russian Academy
of Sciences, Akad. Arbuzov St. 8, Kazan 420088, Russia; microbi@iopc.ru (A.V.);
aplyubina@gmail.com (A.L.)

[4] Institute of Biochemistry and Physiology of Plants and Microorganisms, Saratov Scientific Centre of the
Russian Academy of Sciences, Entuziastov Ave. 13, Saratov 410049, Russia;
tsivileva_o@ibppm.ru

[5] V. A. Negovsky Research Institute of General Reanimatology, Russian Academy of Medical Sciences,
Petrovka St. 25/2, Moscow 107031, Russia; artem_kuzovlev@fnkcrr.ru

[6] School of Pharmacy, Hangzhou Normal University, Yuhangtan, 2318, Hangzhou 310030, China; yi.wang1122@hznu.edu.cn

[7] Department of Pharmacology, Bashkir State Medical University, Lenin St. 3, Ufa 450008, Russia

[8] G. A. Razuvaev Institute of Organometallic Chemistry, RAS, Tropinin Str. 49, Nizhny Novgorod 603950,
Russia; zhiganshinae@mail.ru (E.R.Z.); mars@iomc.ras.ru (M.V.A.),

* Correspondence: abogdanov@inbox.ru (A.V.B.); avsamorodov@gmail.com (A.V.S.);
Tel.: +7-843-272-7384 (A.V.B.); +7-905-006-0385 (A.V.S.)

† These authors contributed equally to this work.

Contents

Copies of NMR spectra S2-S52

Copies of NMR spectra

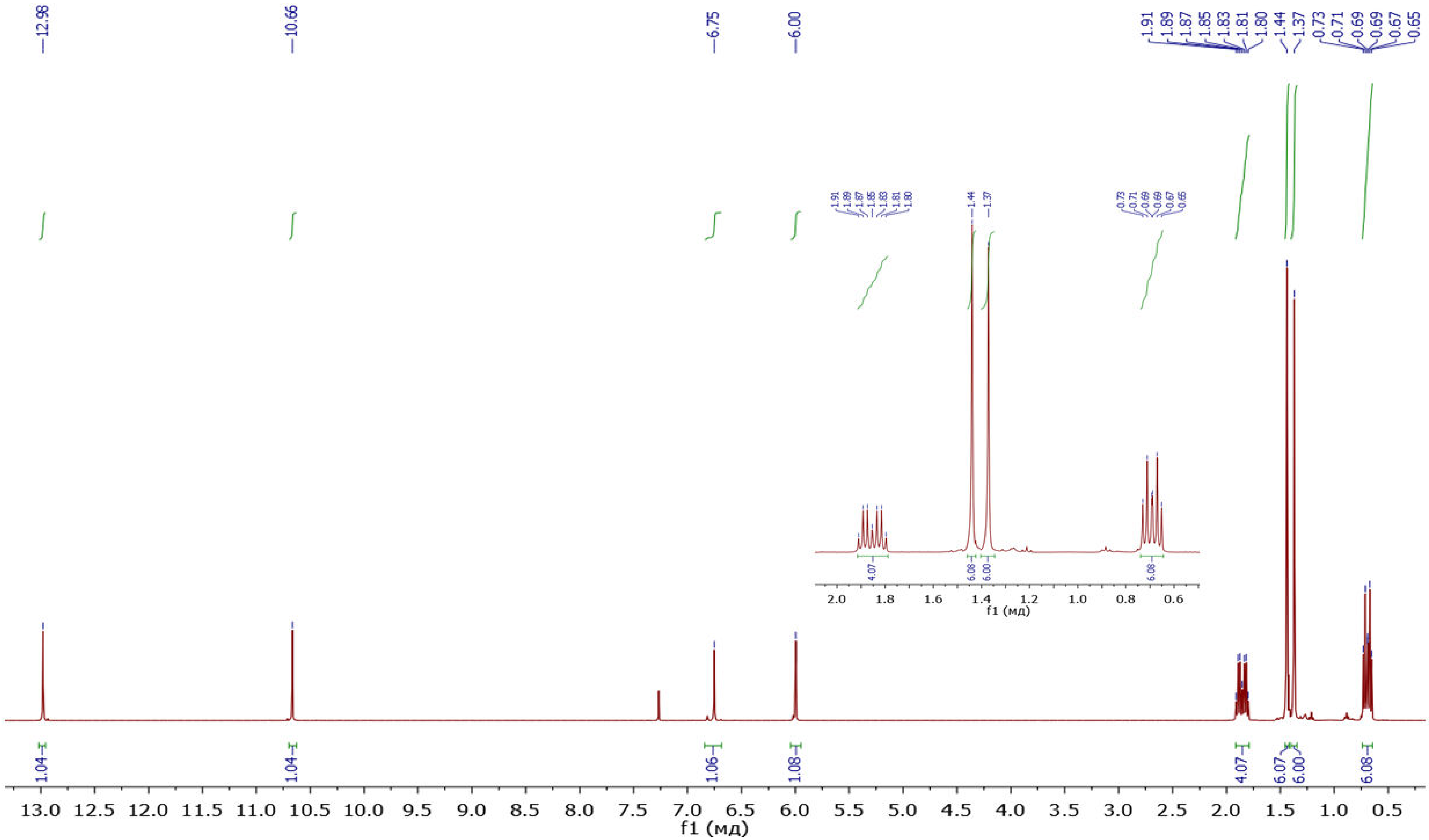

Fig. S1. ^{1}H NMR spectrum of compound **2** (400 MHz, $CDCl_3$)

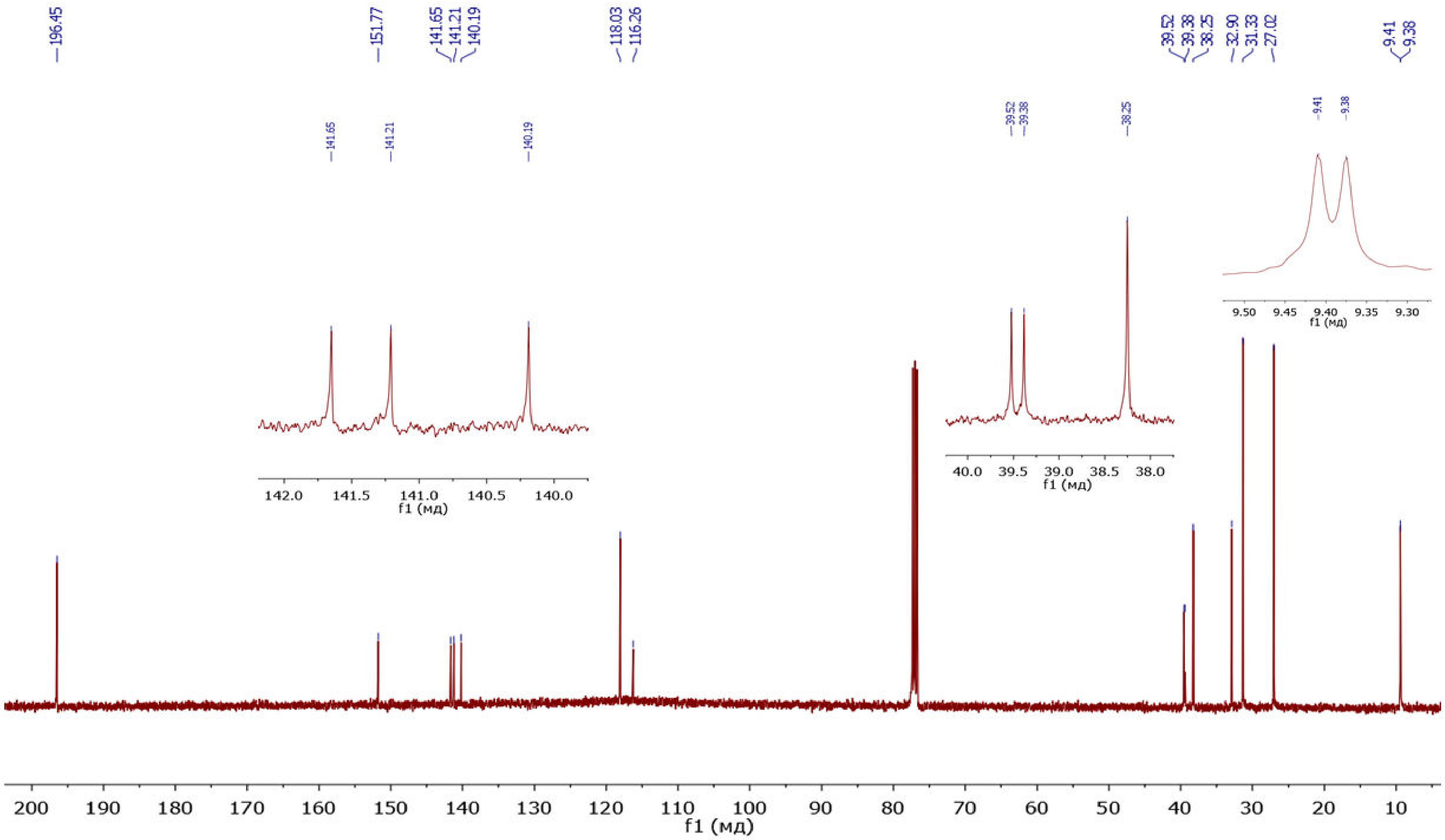

Fig. S2. ^{13}C-{^{1}H} NMR spectrum of compound **2** (101 MHz, $CDCl_3$)

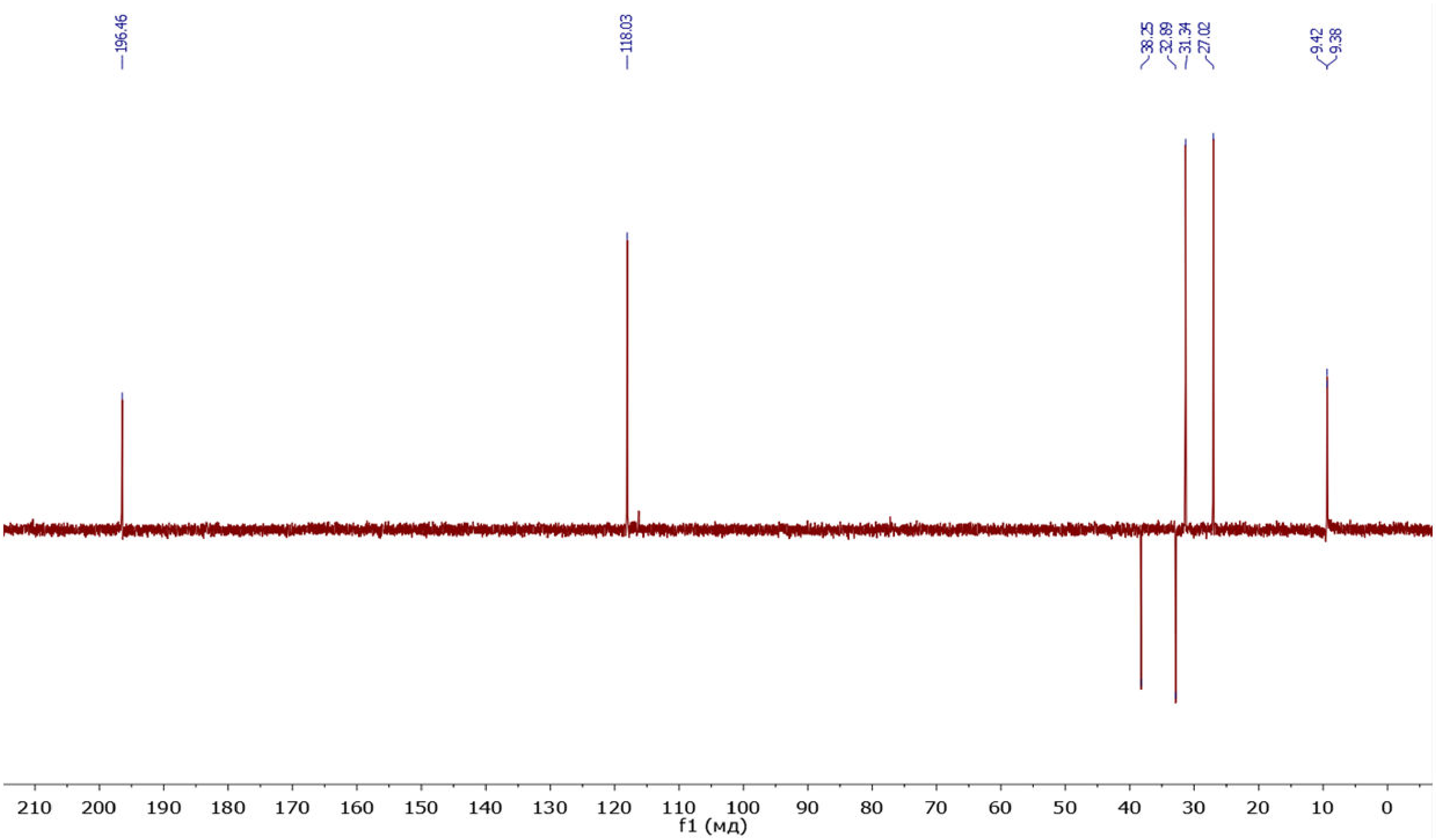

Fig. S3. ^{13}C (dept) NMR spectrum of compound **2** (101 MHz, $CDCl_3$)

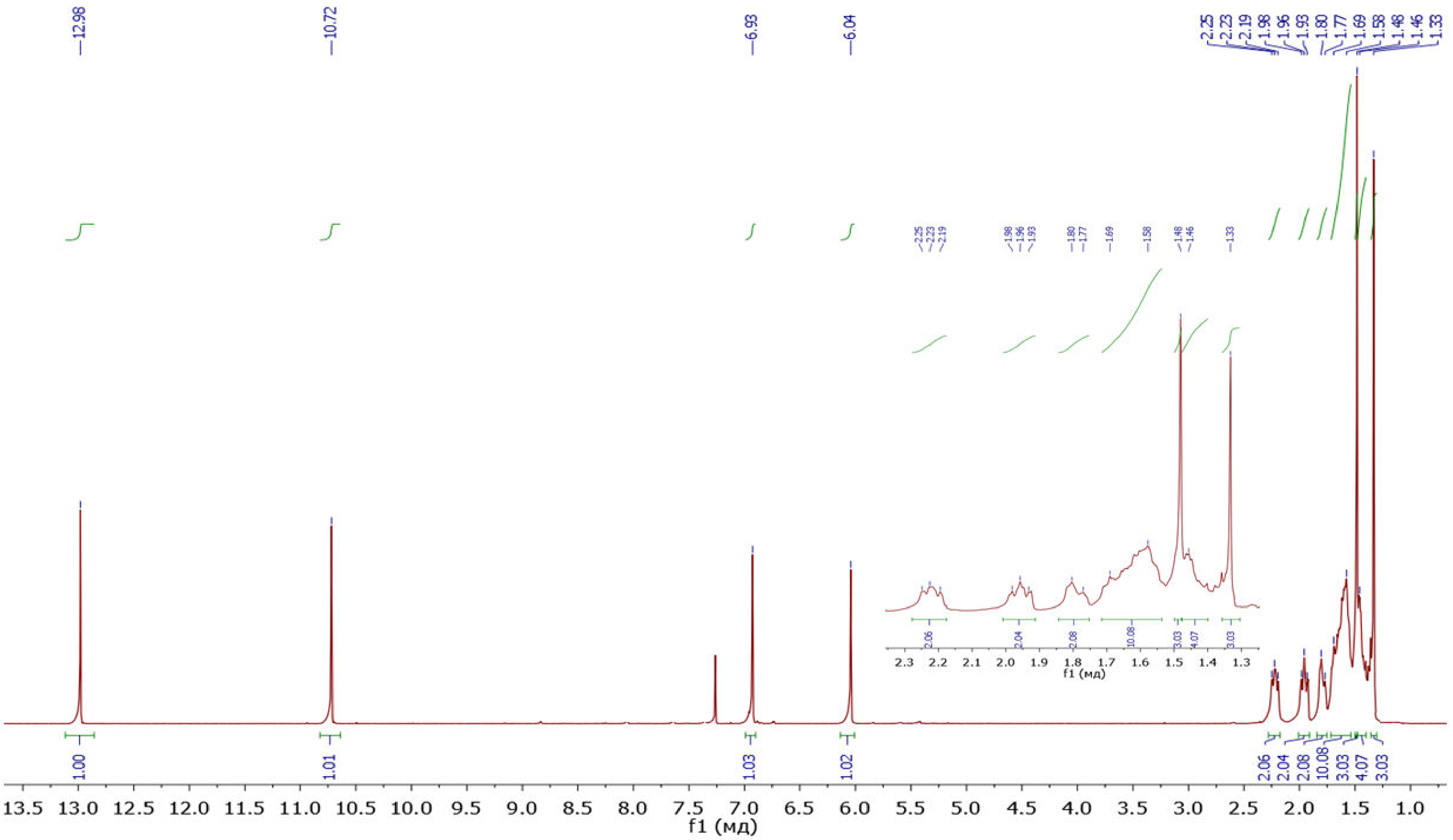

Fig. S4. ^1H NMR spectrum of compound **4** (400 MHz, $CDCl_3$)

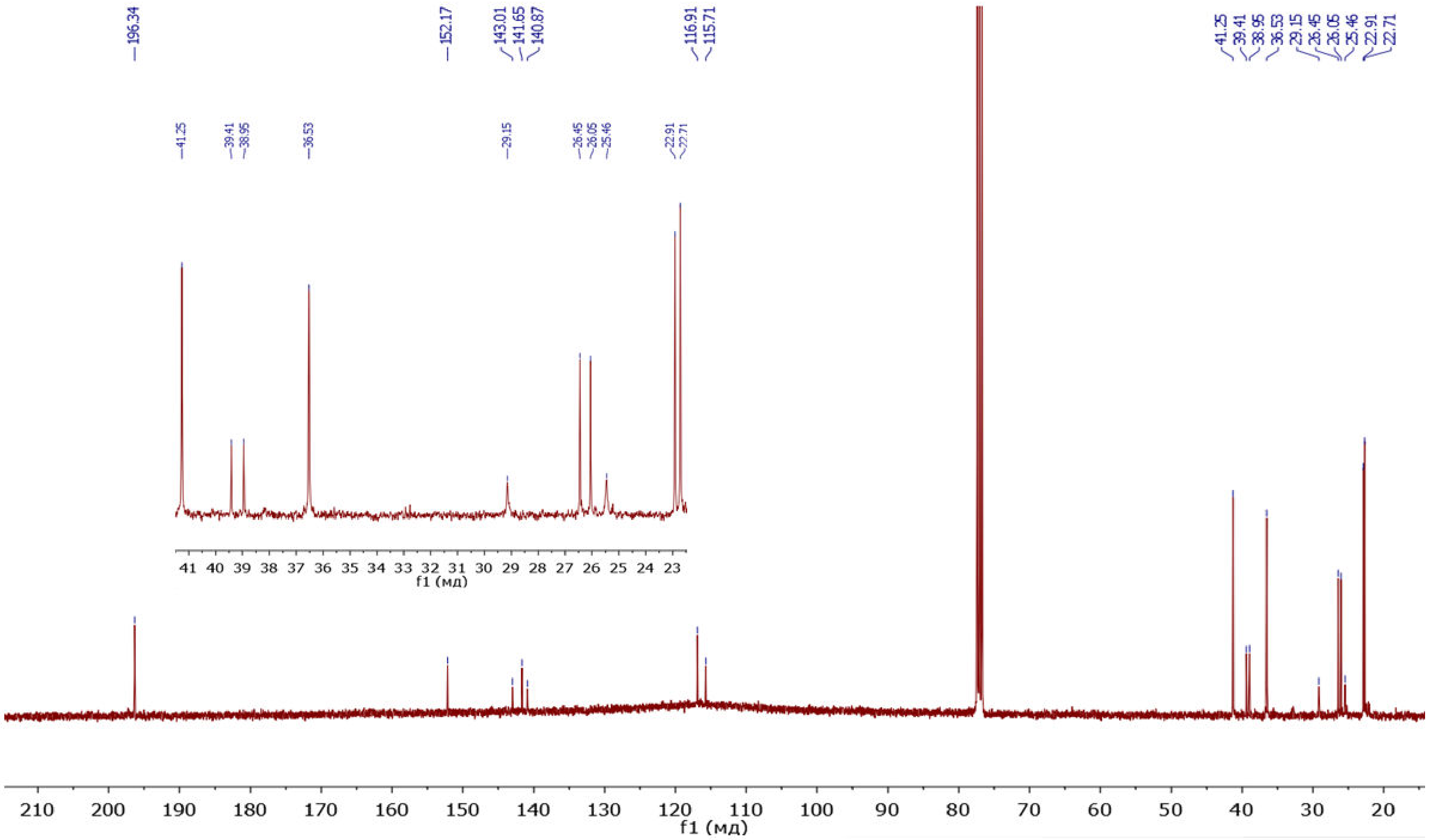

Fig. S5. ^{13}C-{^1H} NMR spectrum of compound **4** (101 MHz, $CDCl_3$)

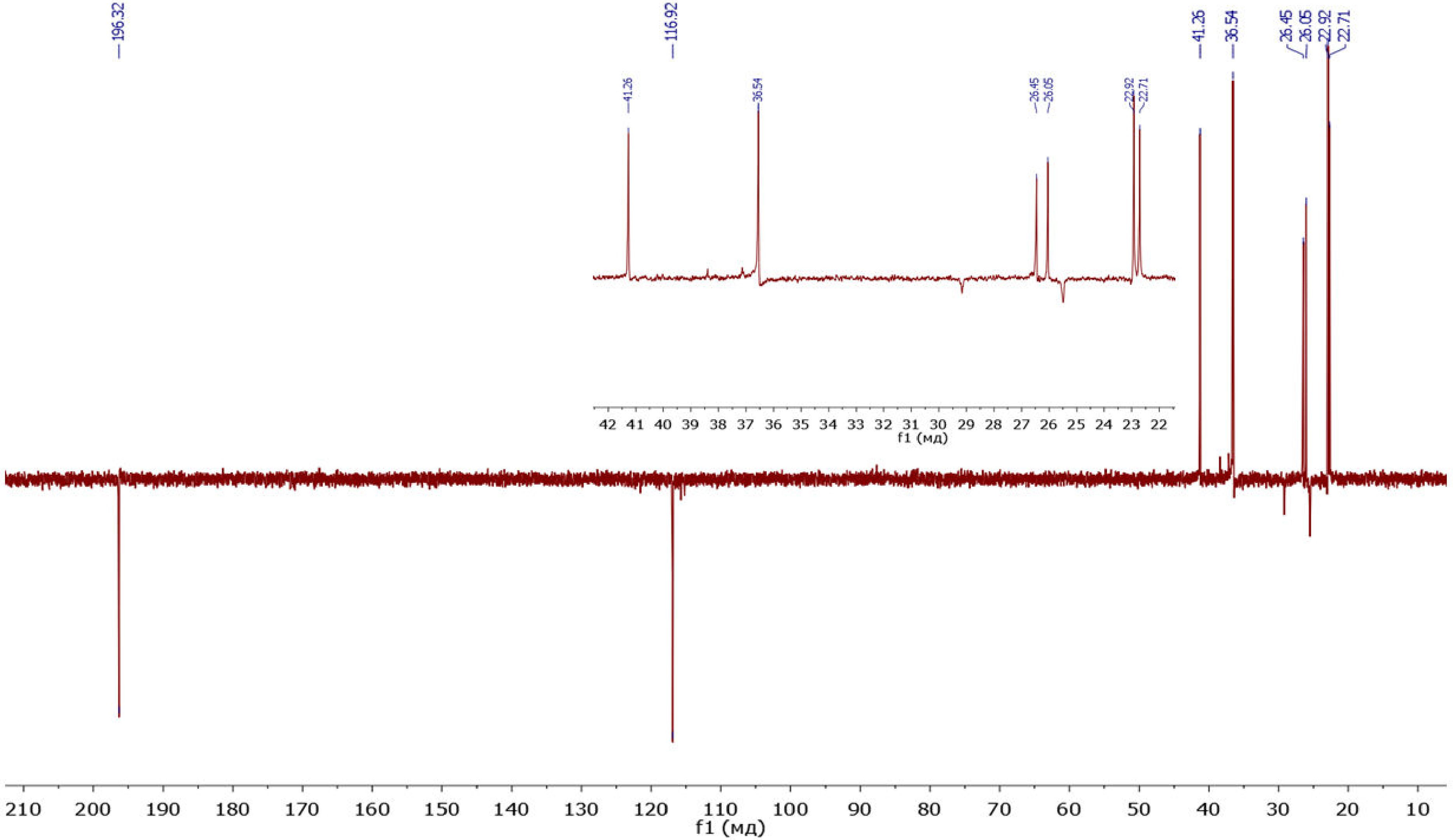

Fig. S6. ^{13}C (dept) NMR spectrum of compound **4** (101 MHz, $CDCl_3$)

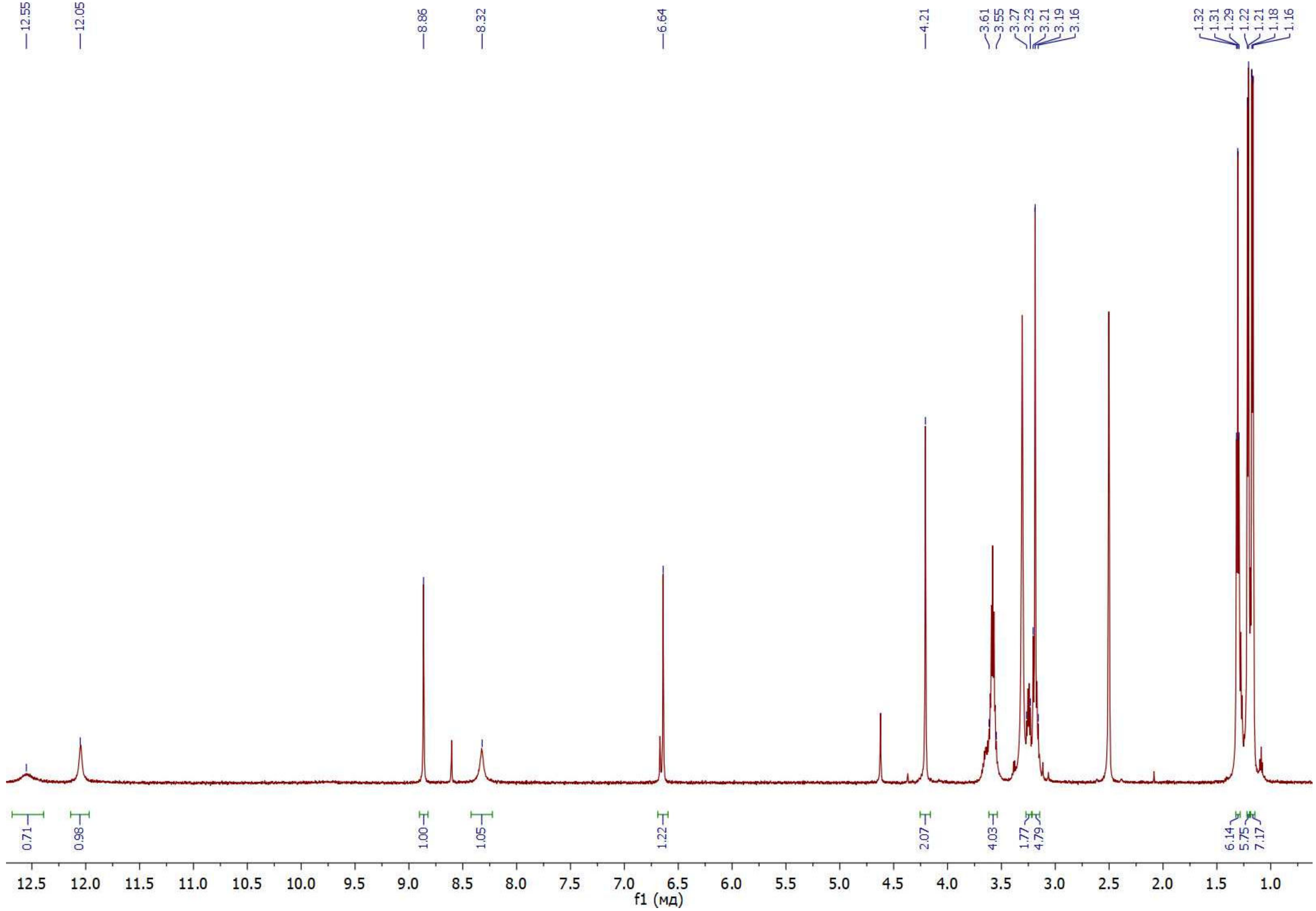

Fig. S7. ^{1}H NMR spectrum of compound **9a** (400 MHz, DMSO-d_6)

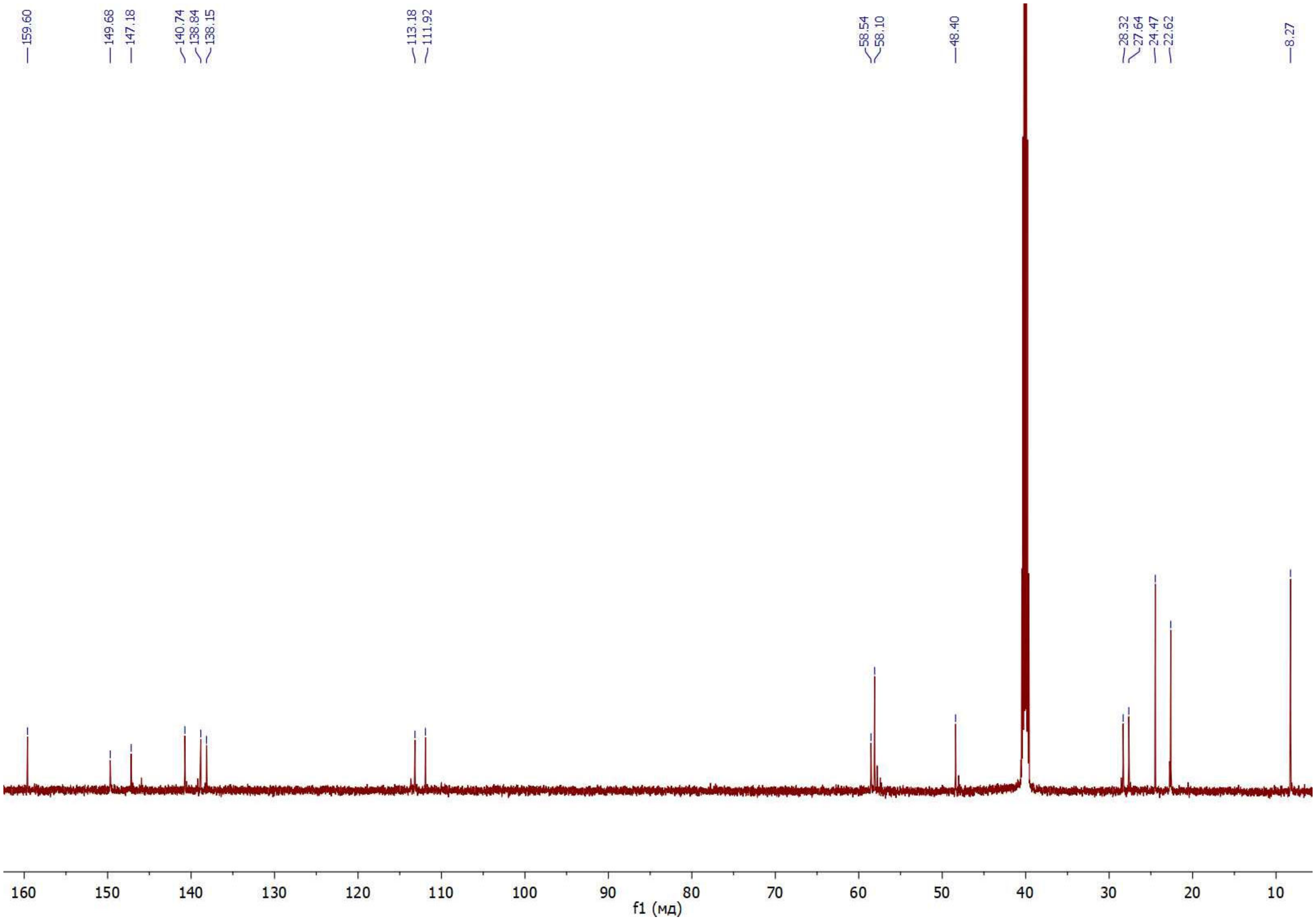

Fig. S8. ^{13}C-{^1H} NMR spectrum of compound **9a** (101 MHz, DMSO-d_6)

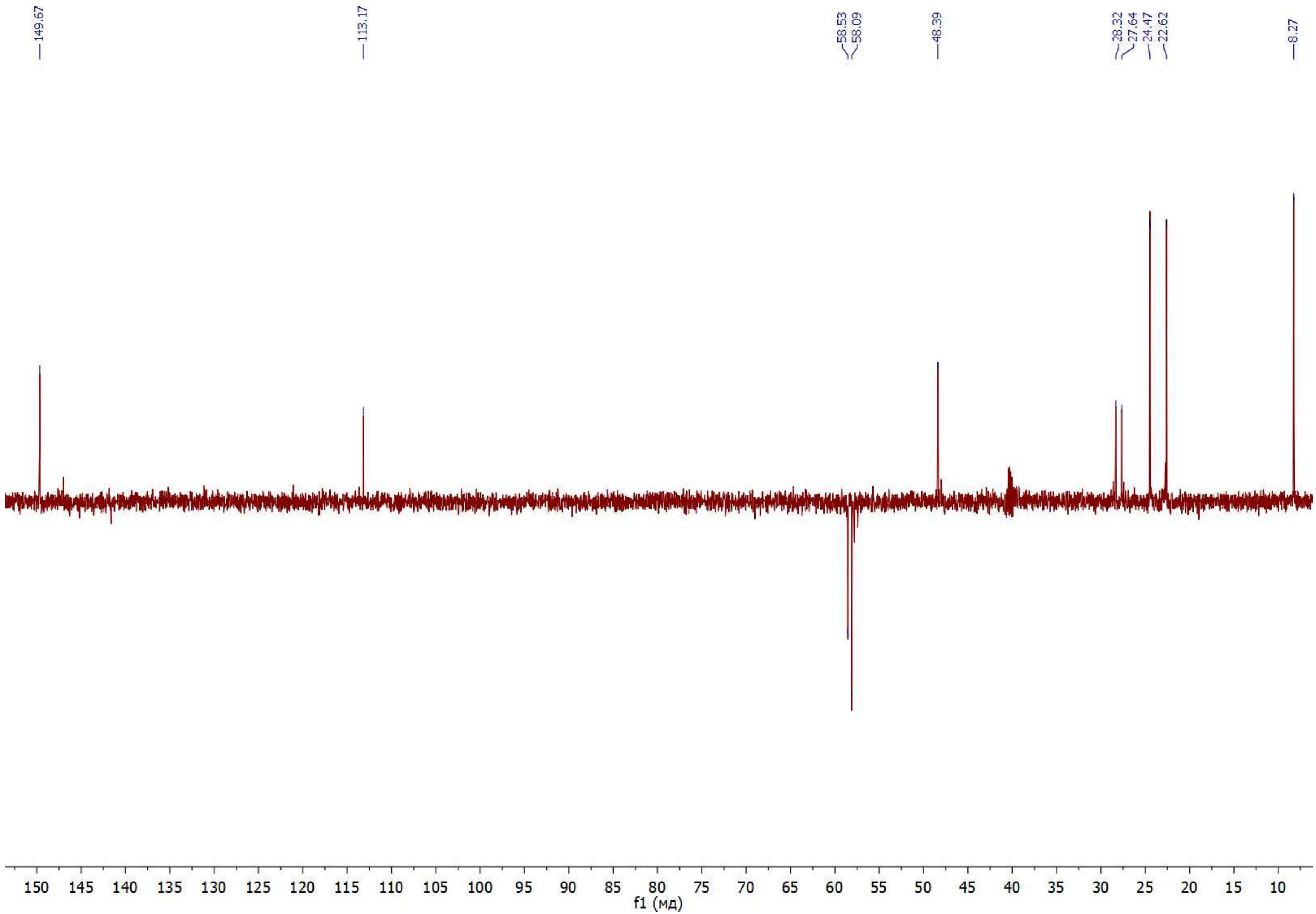

Fig. S9. ^{13}C (dept) NMR spectrum of compound **9a** (101 MHz, DMSO-d_6)

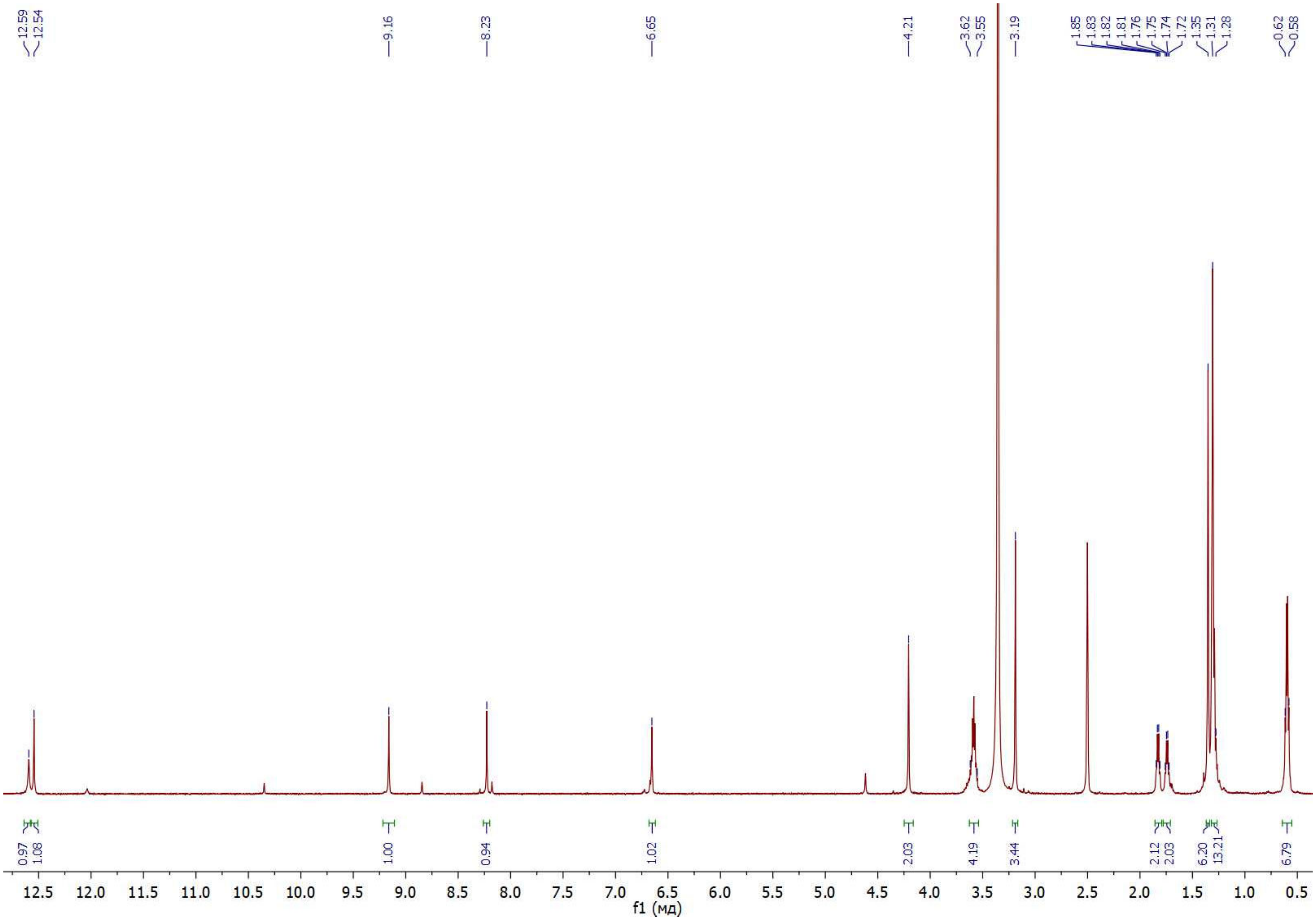

Fig. S10. ^{1}H NMR spectrum of compound **9b** (600 MHz, DMSO-d_6)

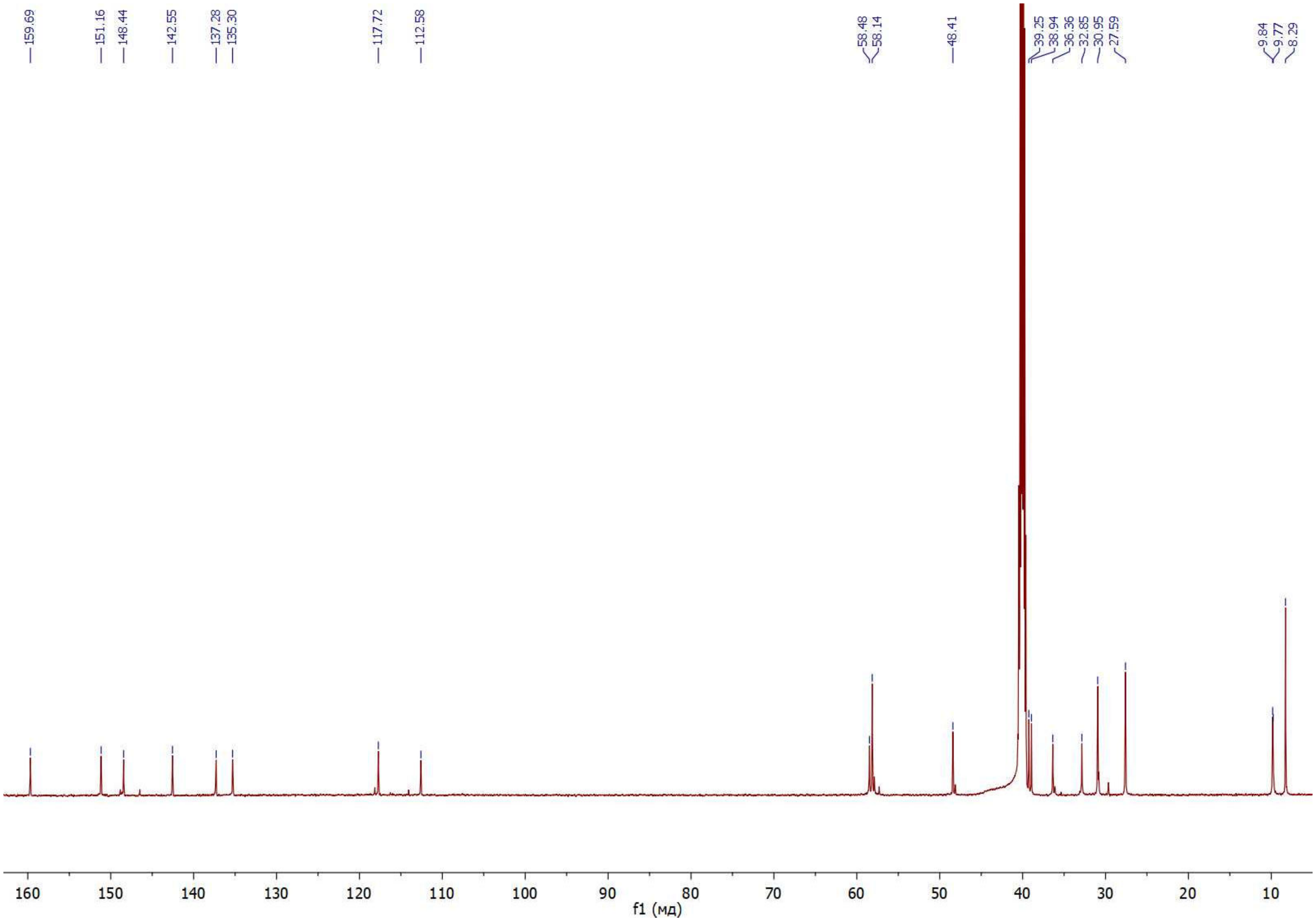

Fig. S11. ^{13}C-{^{1}H} NMR spectrum of compound **9b** (151 MHz, DMSO-d_6)

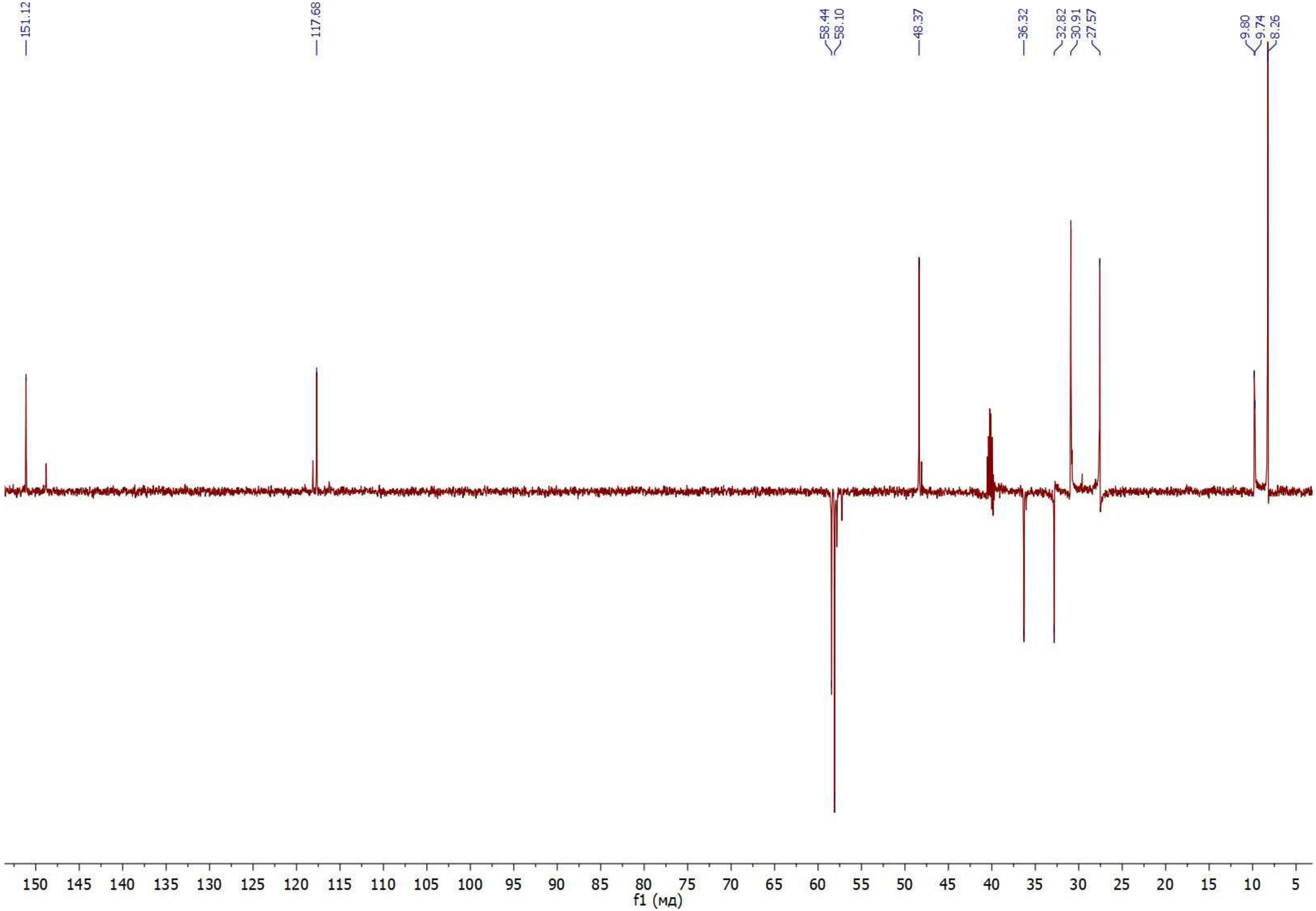

Fig. S12. ^{13}C (dept) NMR spectrum of compound **9b** (151 MHz, DMSO-d_6)

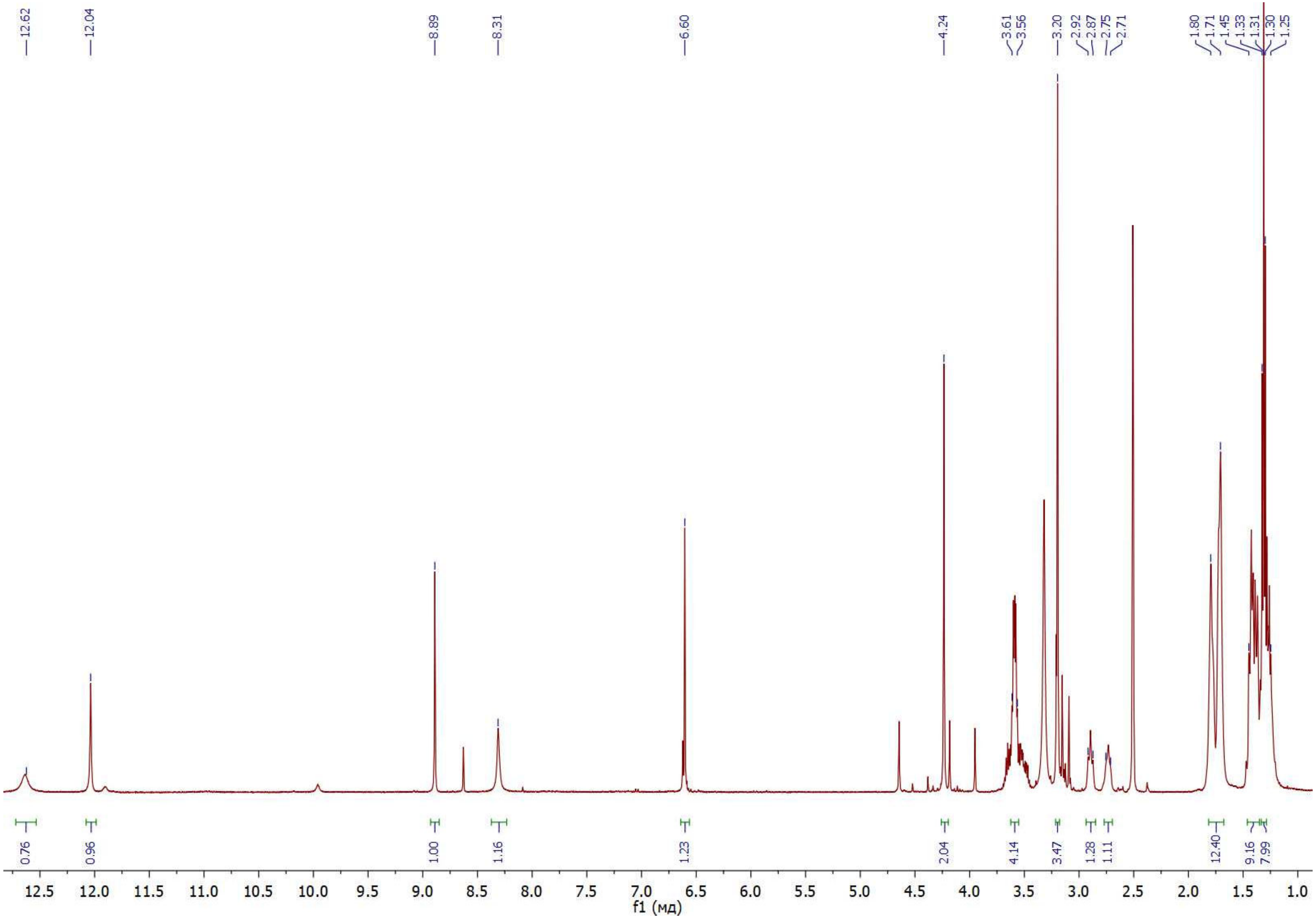

Fig. S13. ^{1}H NMR spectrum of compound **9c** (400 MHz, DMSO-d_6)

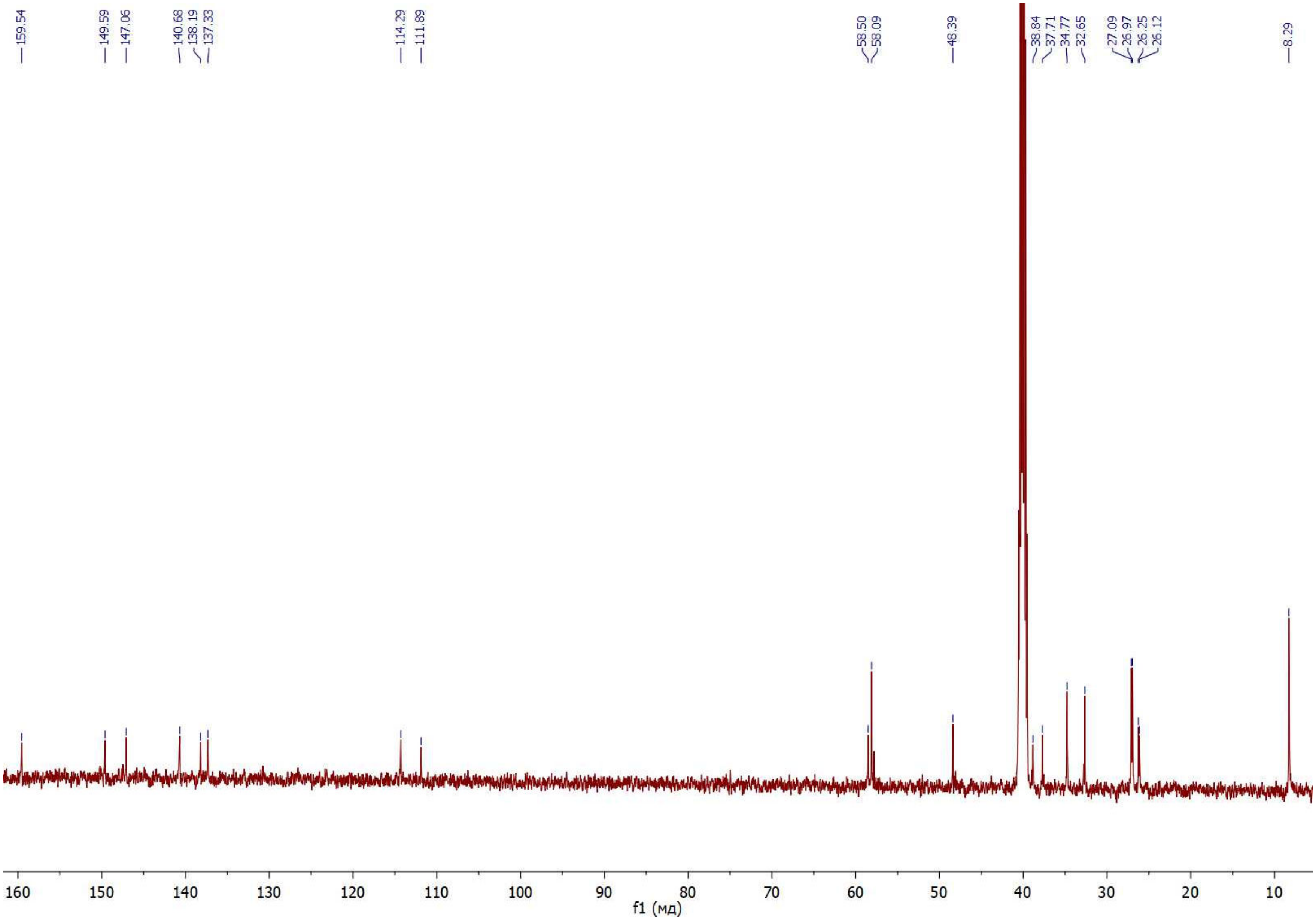

Fig. S14. ^{13}C-{^{1}H} NMR spectrum of compound **9c** (101 MHz, DMSO-d_6)

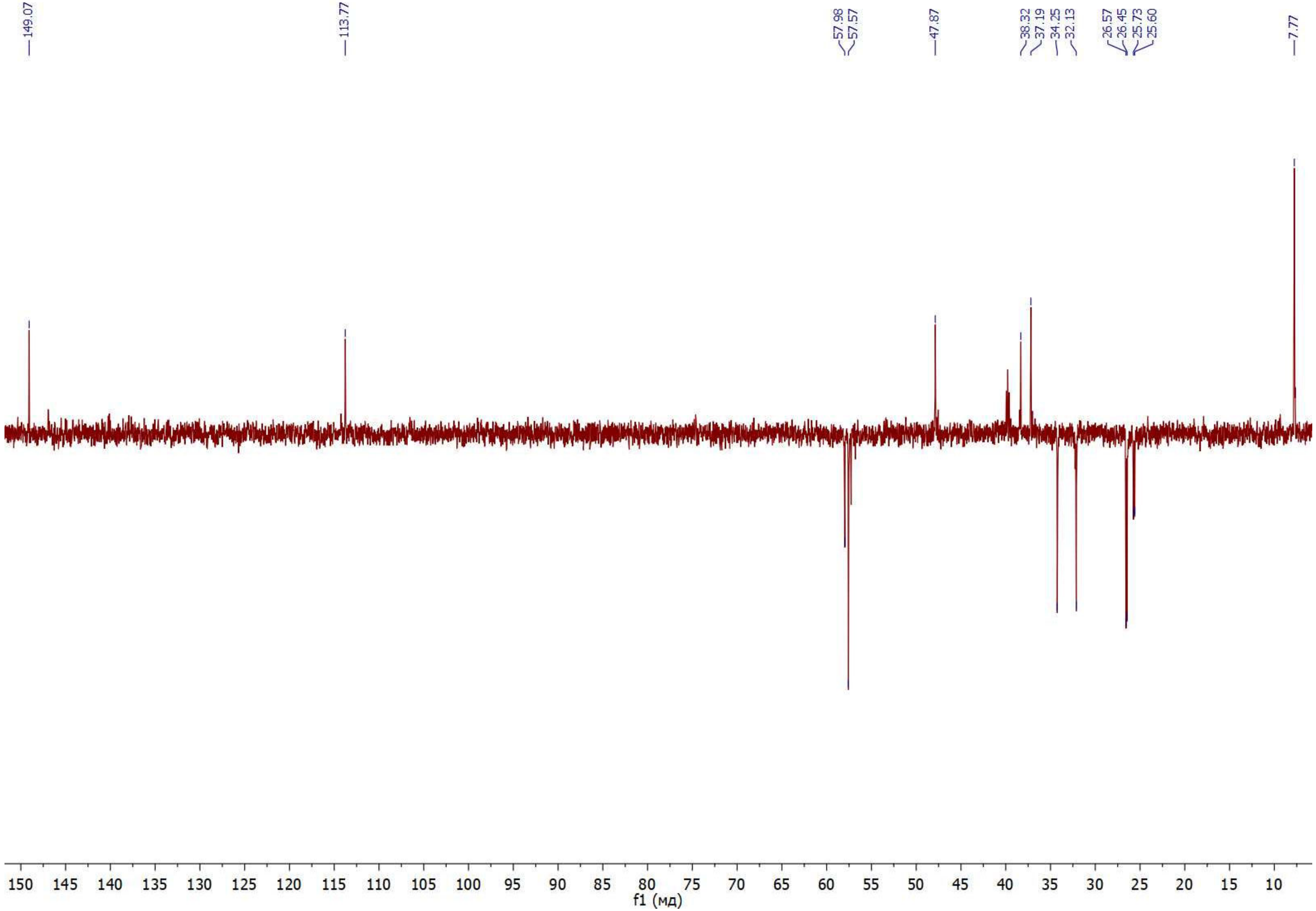

Fig. S15. ^{13}C (dept) NMR spectrum of compound **9c** (101 MHz, DMSO-d_6)

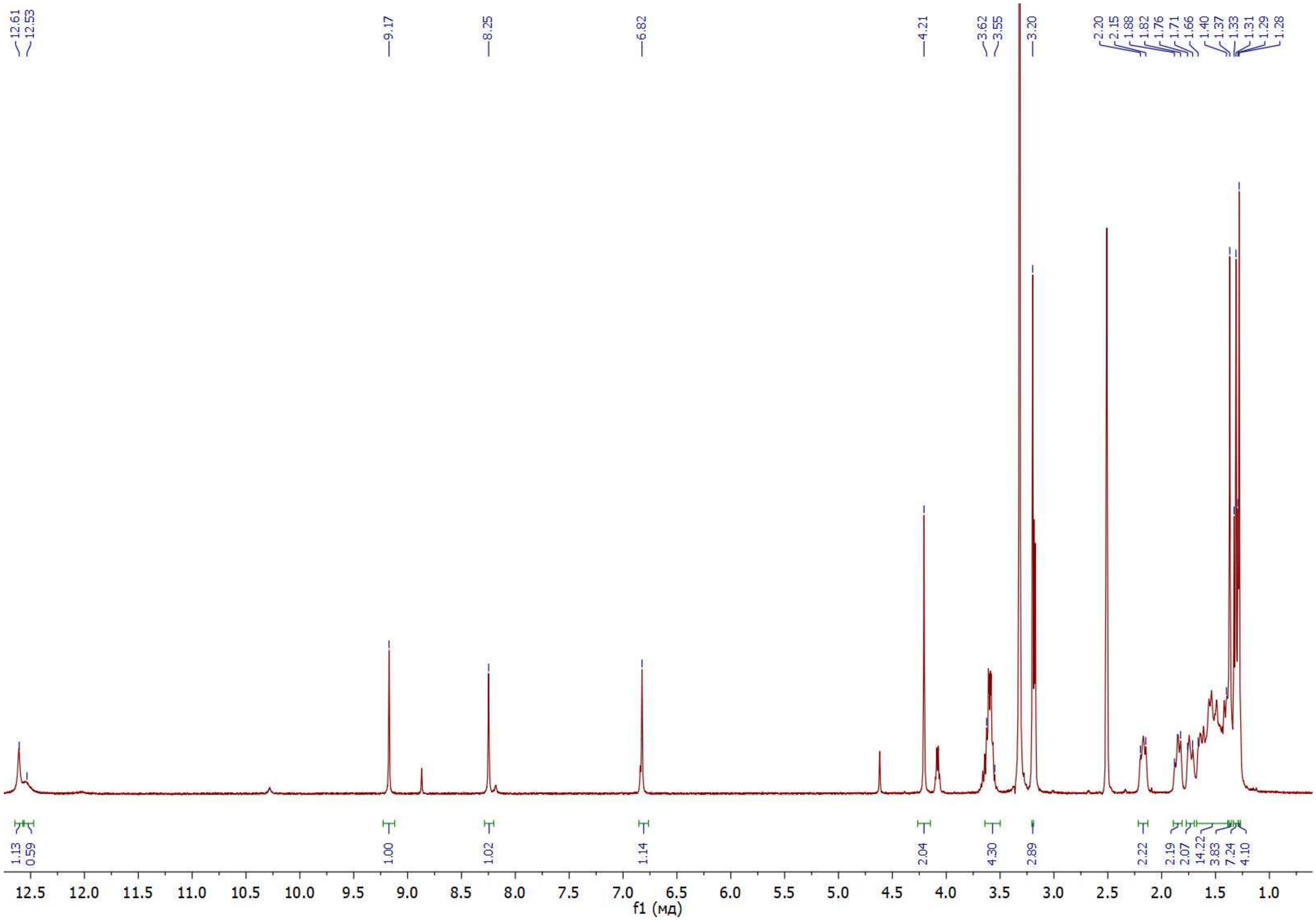

Fig. S16. ^{1}H NMR spectrum of compound **9d** (400 MHz, DMSO-d_6)

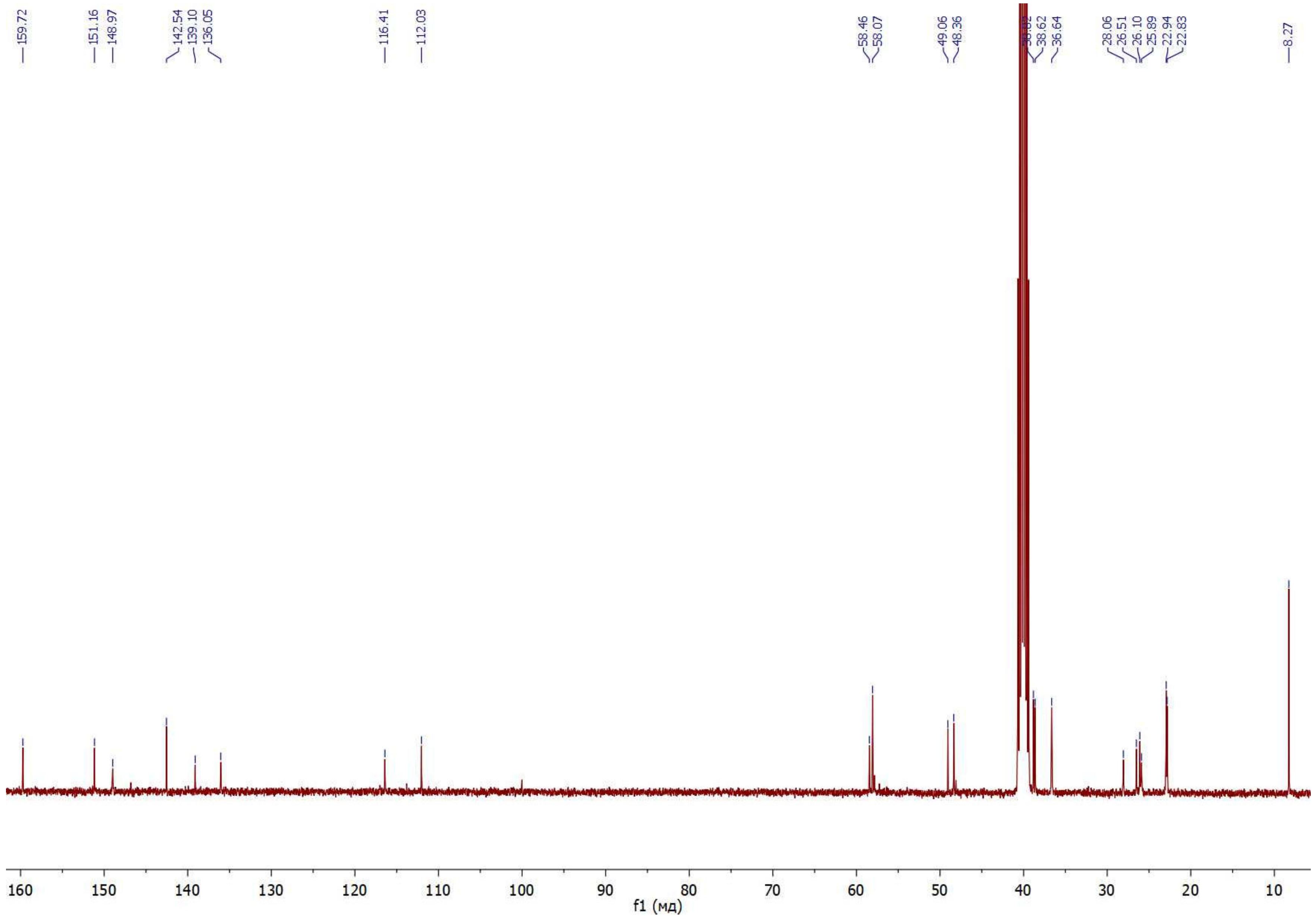

Fig. S17. ^{13}C-{^{1}H} NMR spectrum of compound **9d** (101 MHz, DMSO-d_6)

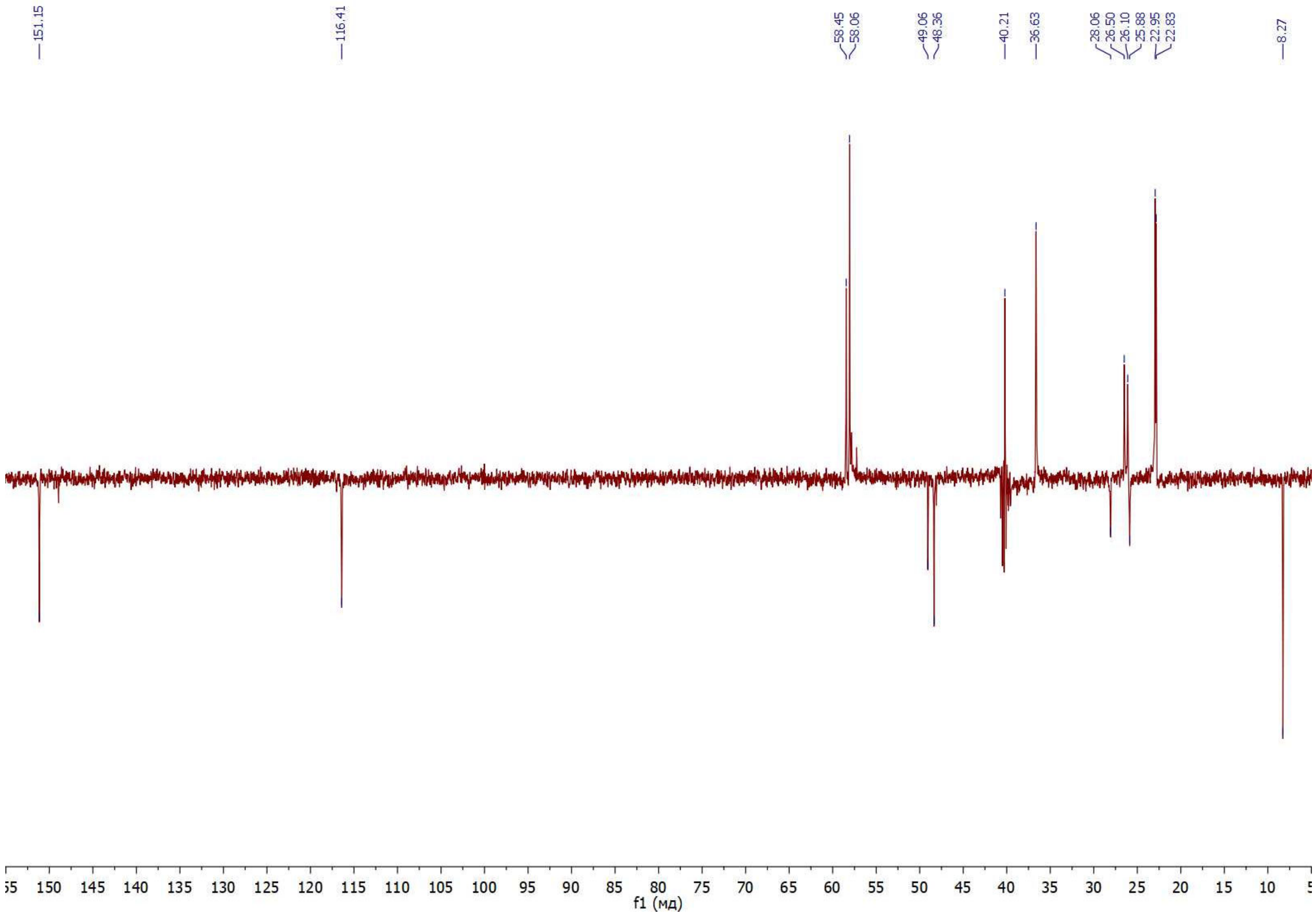

Fig. S18. ^{13}C (dept) NMR spectrum of compound **9d** (101 MHz, DMSO-d_6)

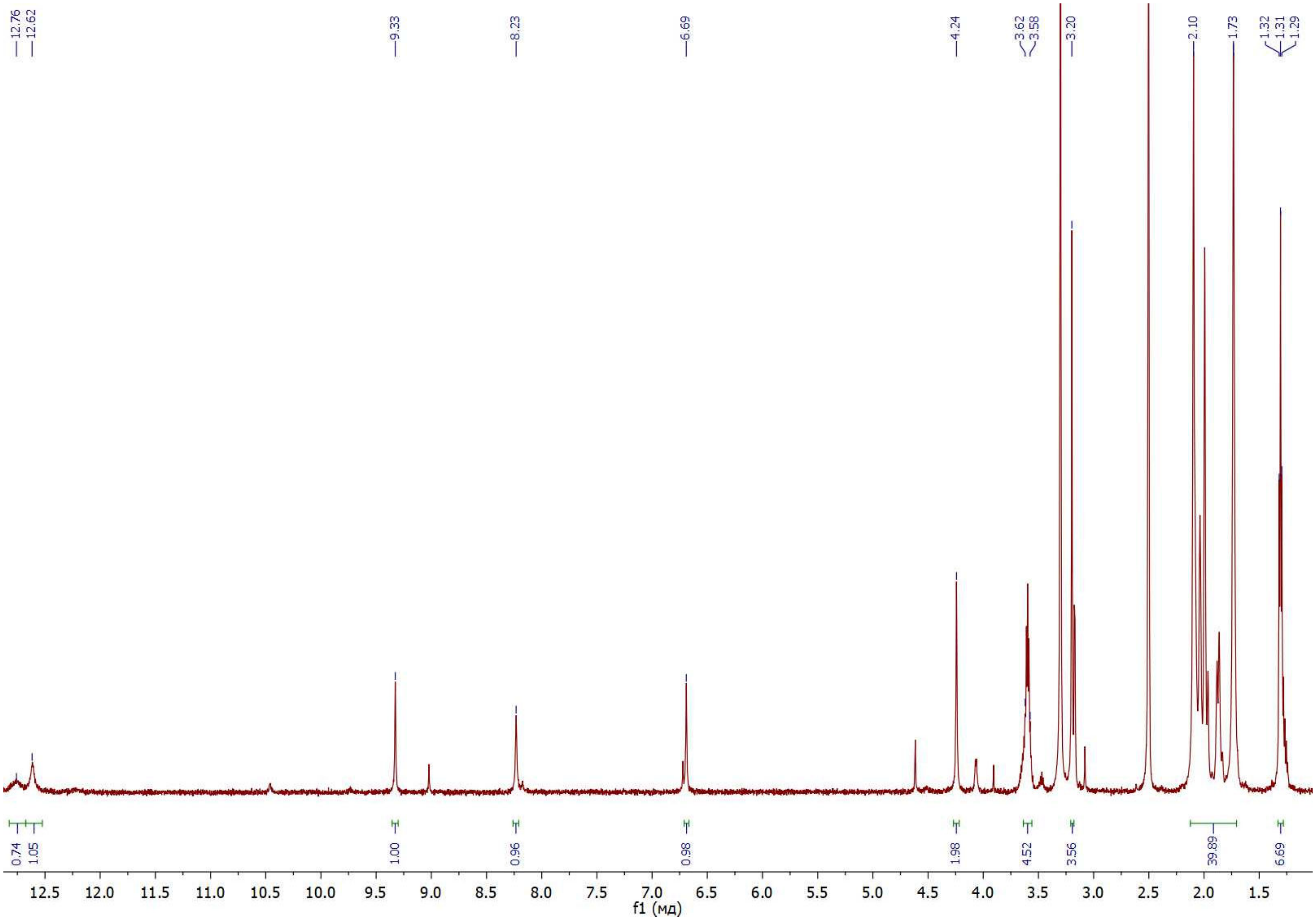

Fig. S19. ^{1}H NMR spectrum of compound **9e** (600 MHz, DMSO-d_6)

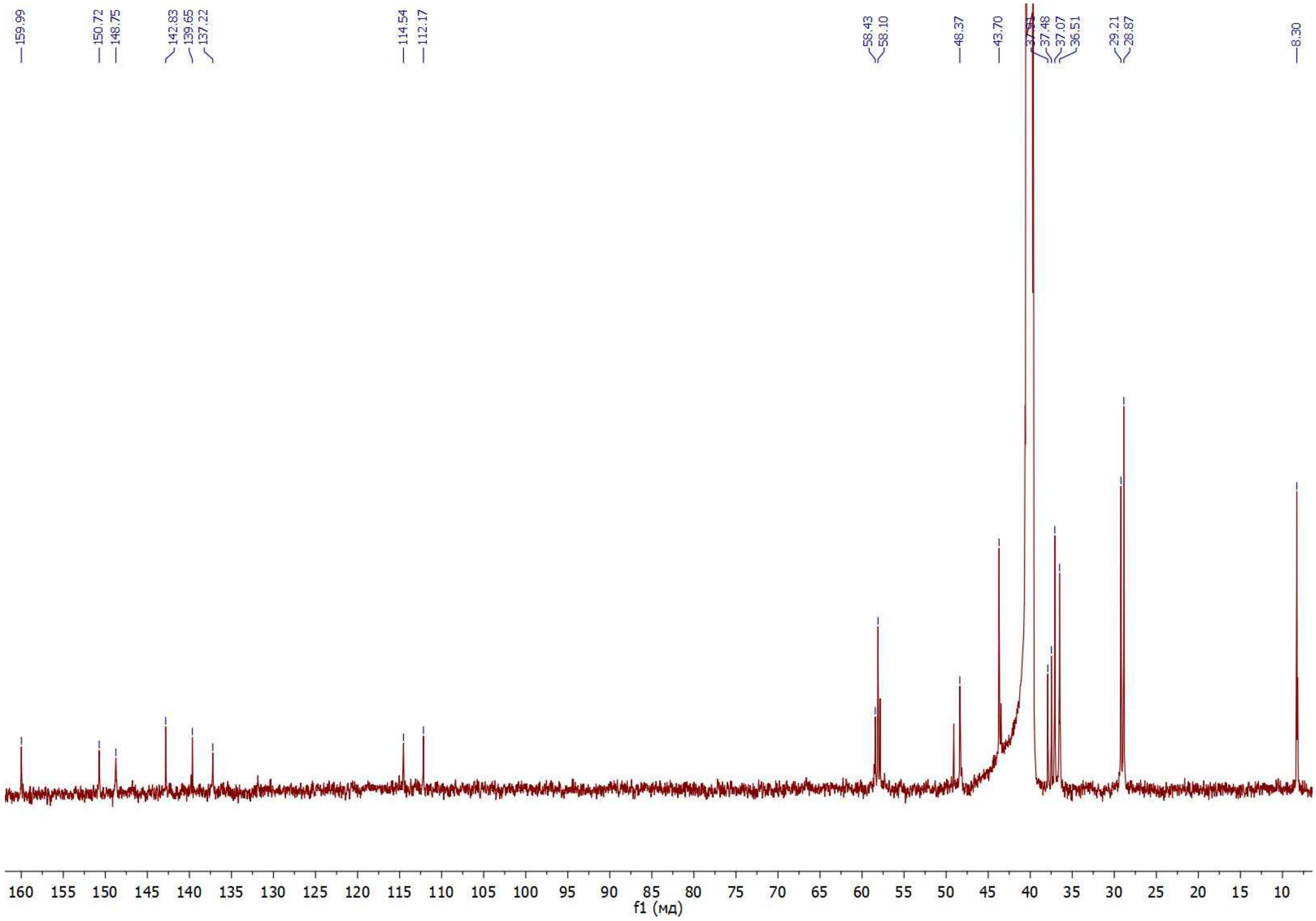

Fig. S20. ^{13}C-{^{1}H} NMR spectrum of compound **9e** (151 MHz, DMSO-d_6)

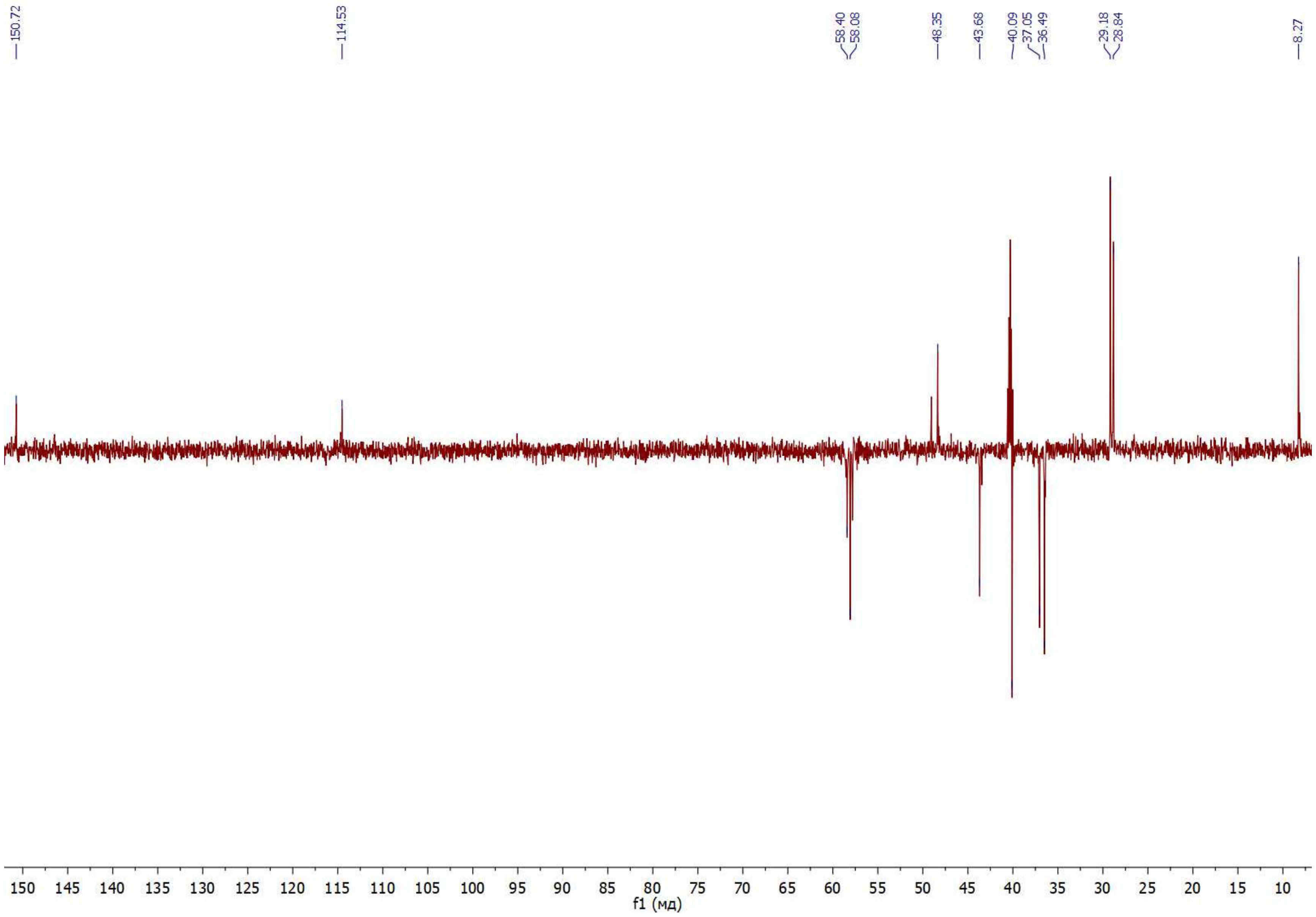

Fig. S21. ^{13}C (dept) NMR spectrum of compound **9e** (151 MHz, DMSO-d_6)

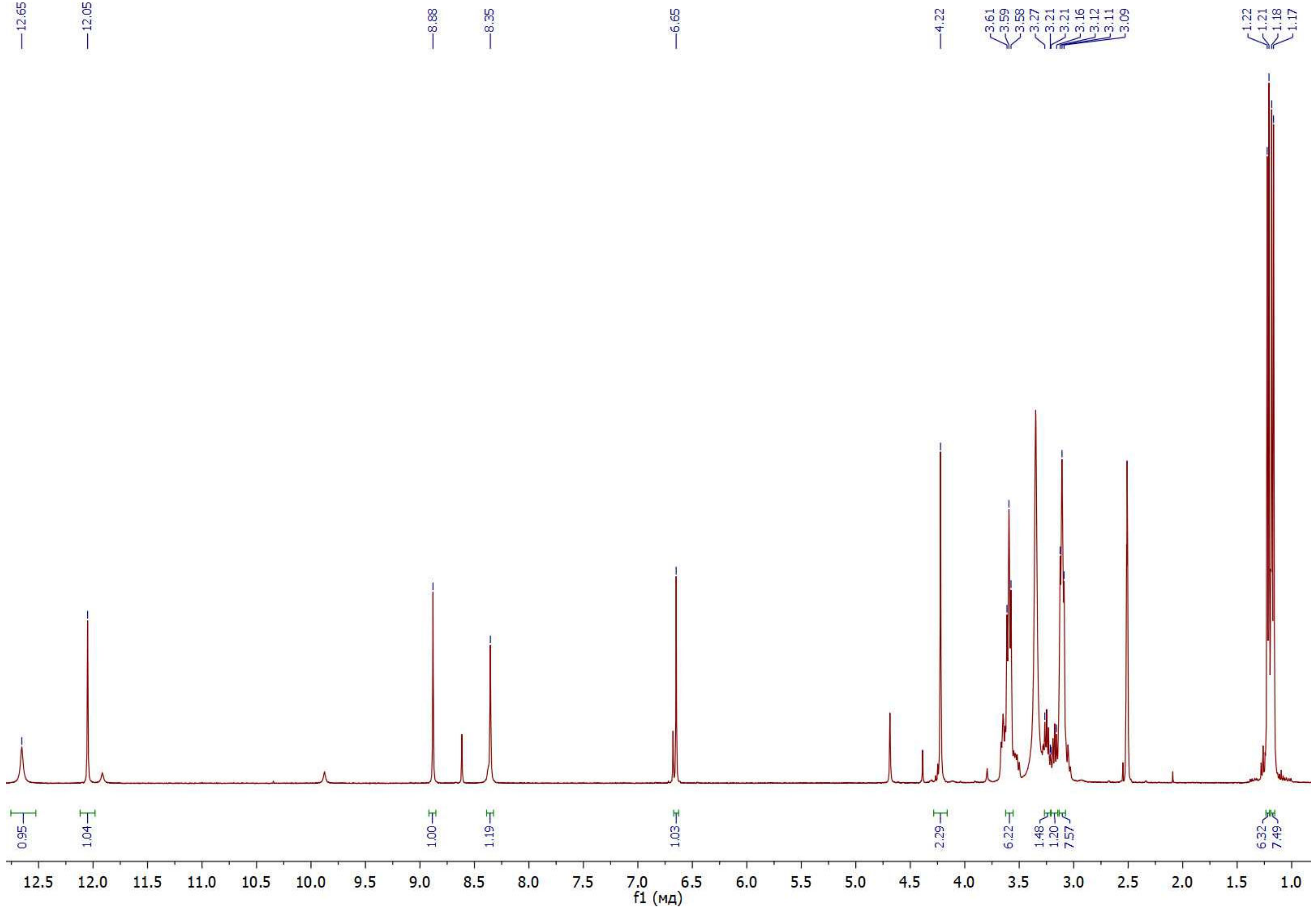

Fig. S22. ^{1}H NMR spectrum of compound **10a** (500 MHz, DMSO-d_6)

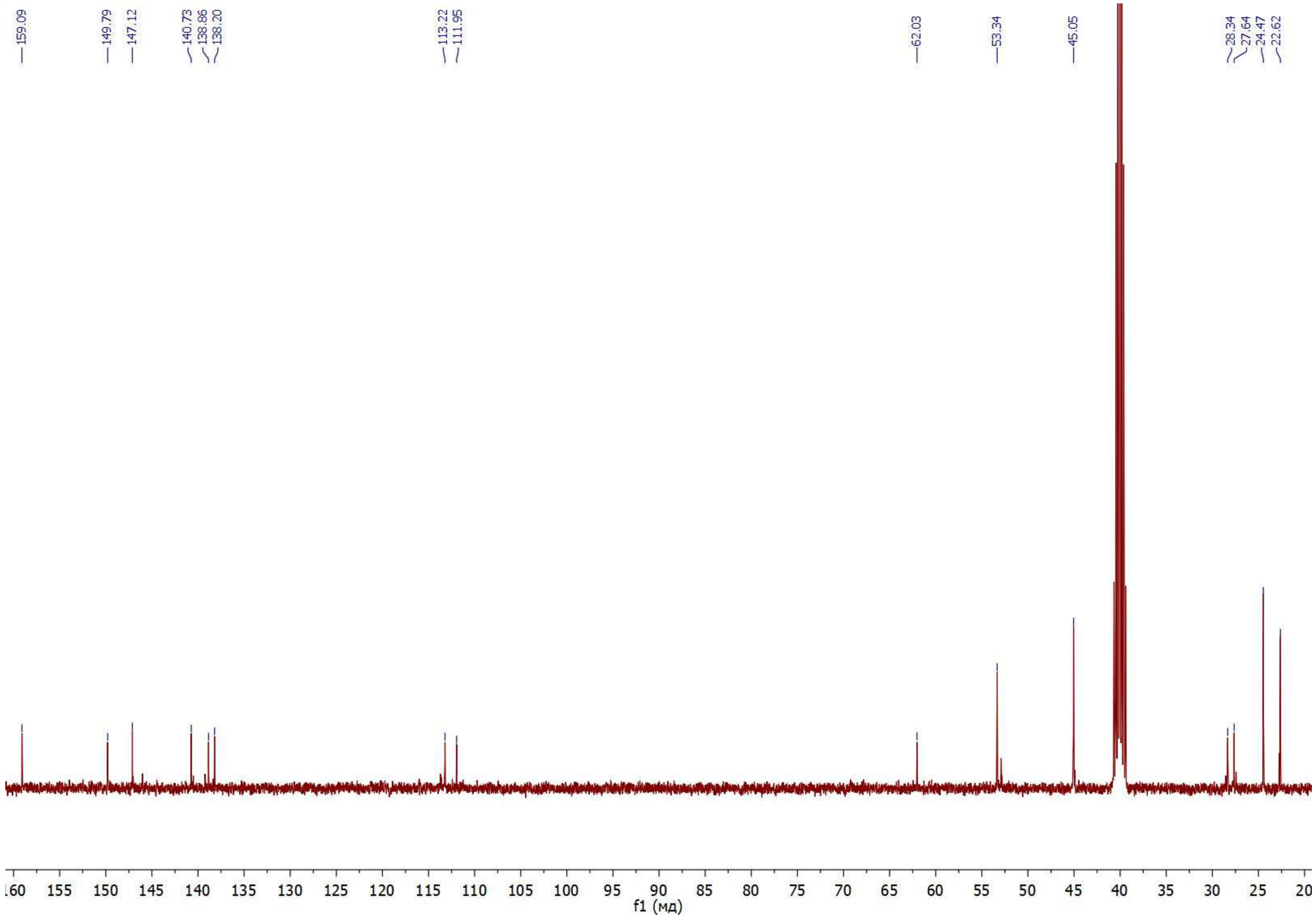

Fig. S23. ^{13}C-{^{1}H} NMR spectrum of compound **10a** (126 MHz, DMSO-d_6)

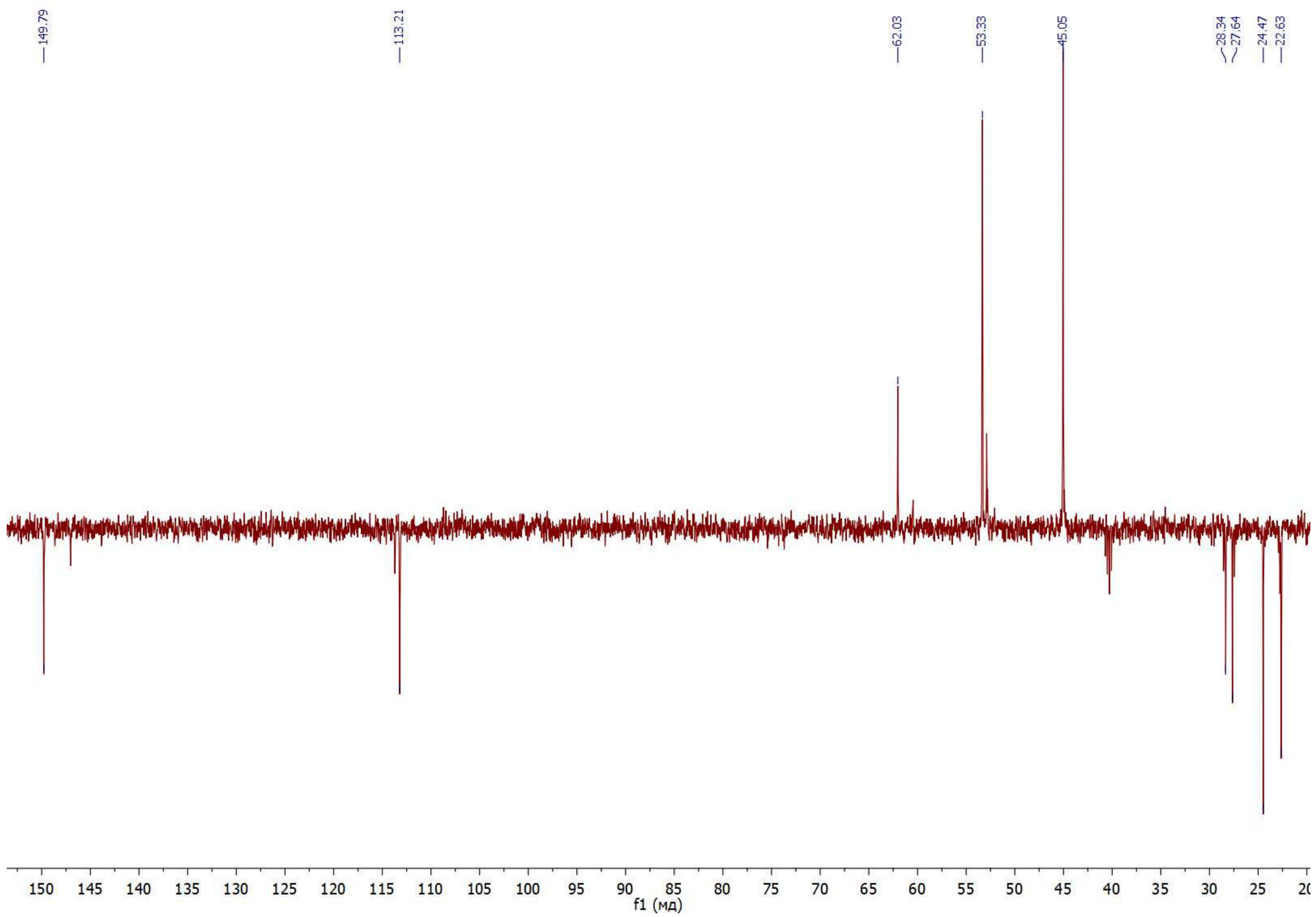

Fig. S24. ^{13}C (dept) NMR spectrum of compound **10a** (126 MHz, DMSO-d_6)

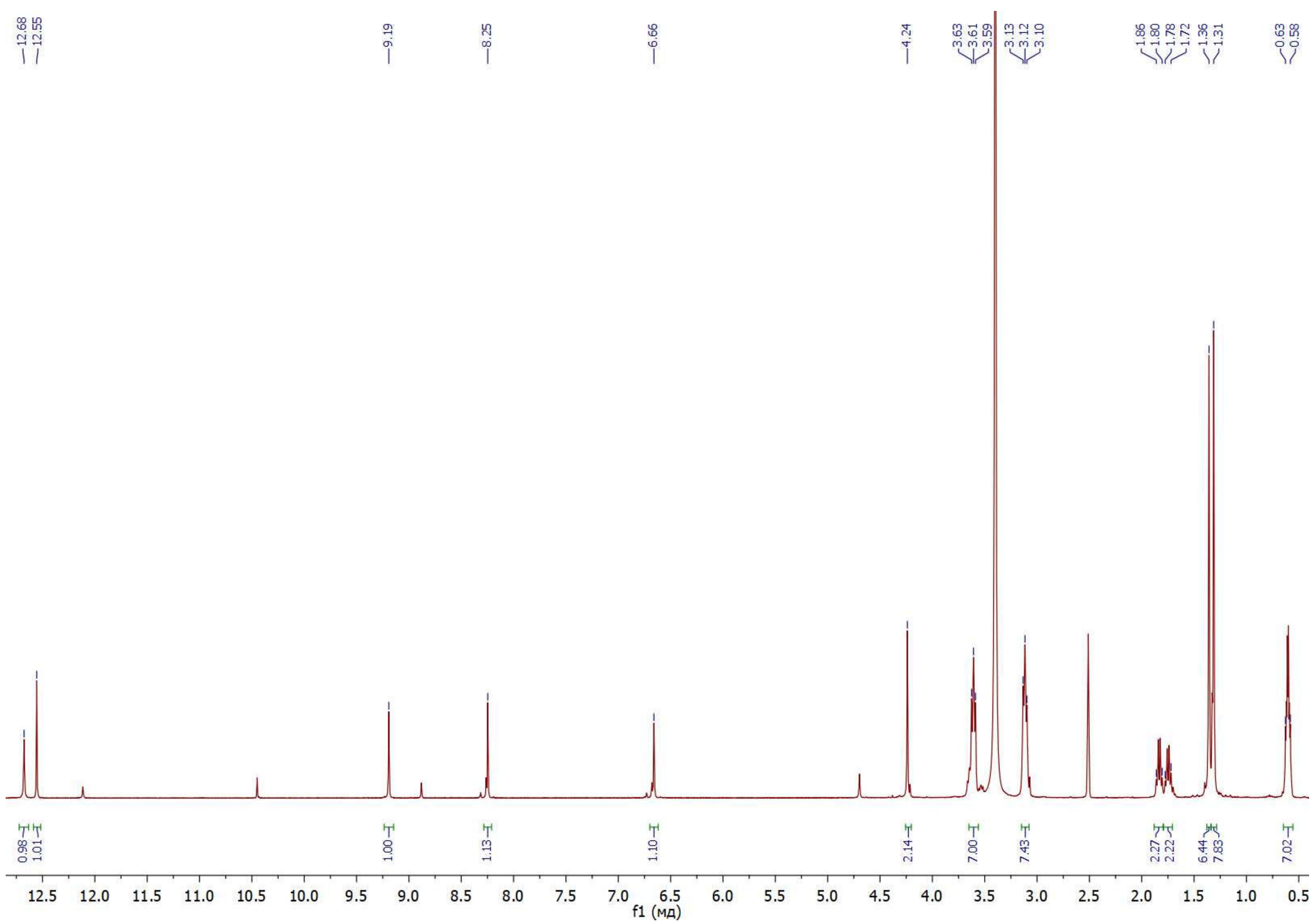

Fig. S25. ^{1}H NMR spectrum of compound **10b** (400 MHz, DMSO-d_6)

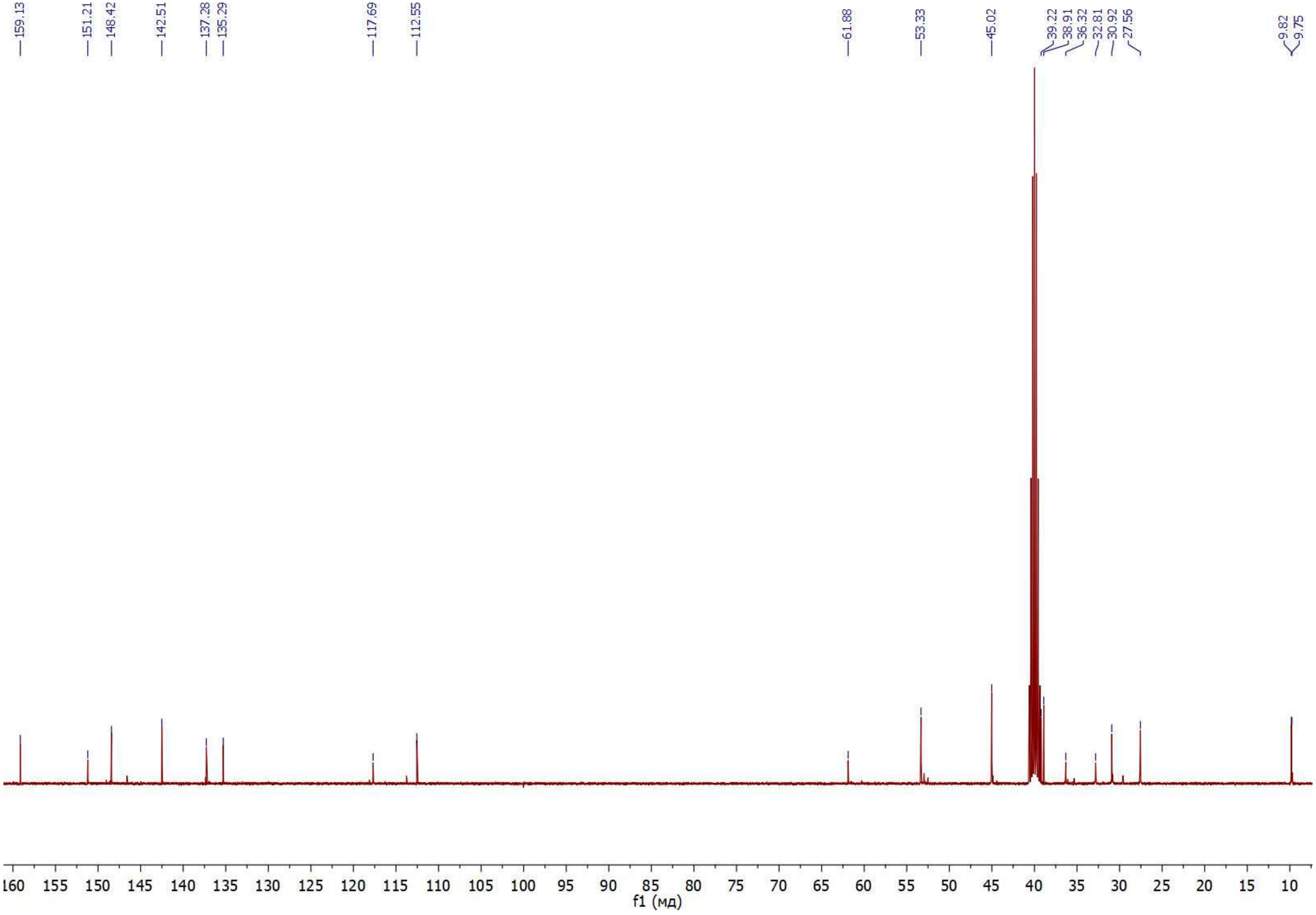

Fig. S26. ^{13}C-{^{1}H} NMR spectrum of compound **10b** (101 MHz, DMSO-d_6)

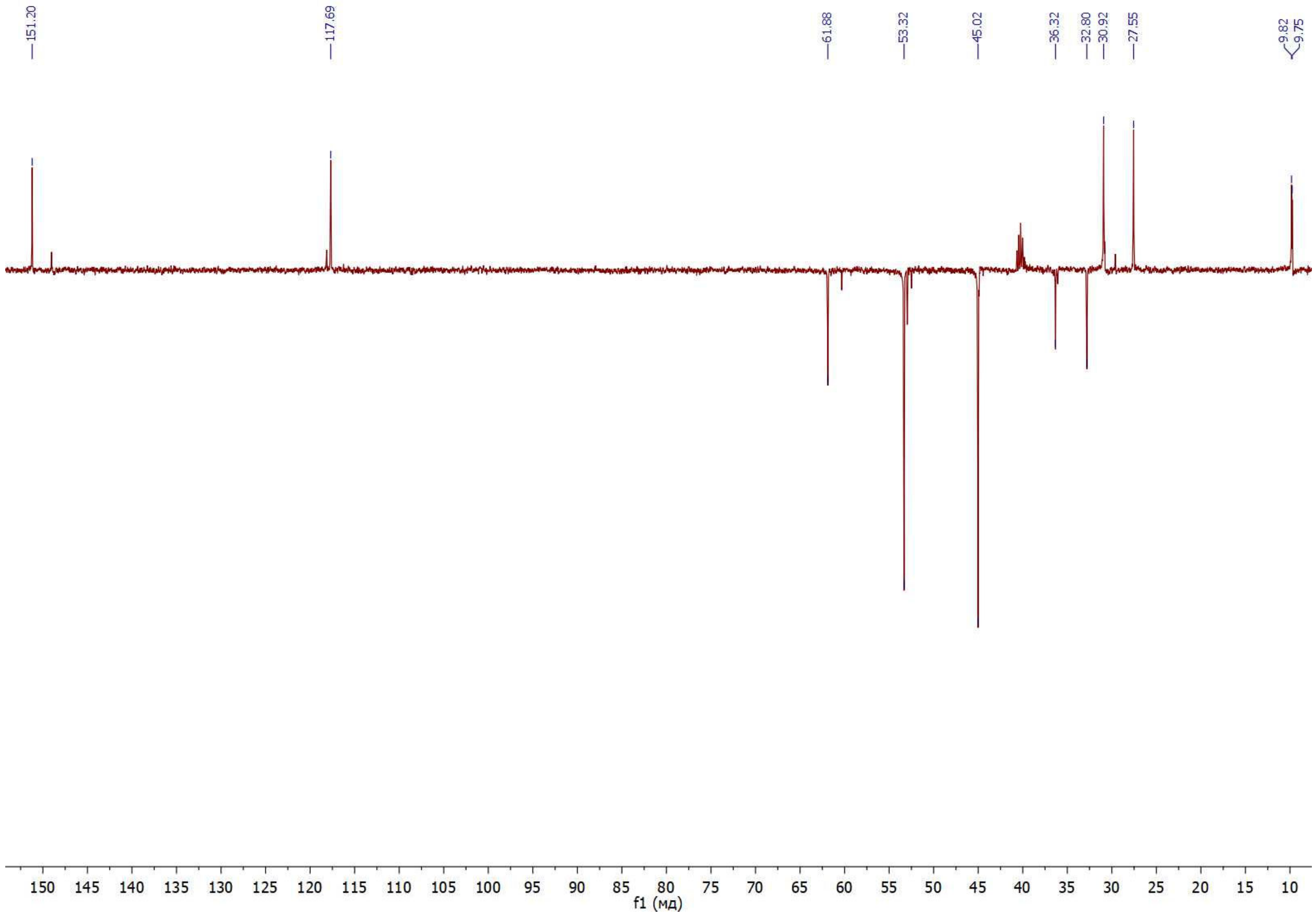

Fig. S27. ^{13}C (dept) NMR spectrum of compound **10b** (101 MHz, DMSO-d_6)

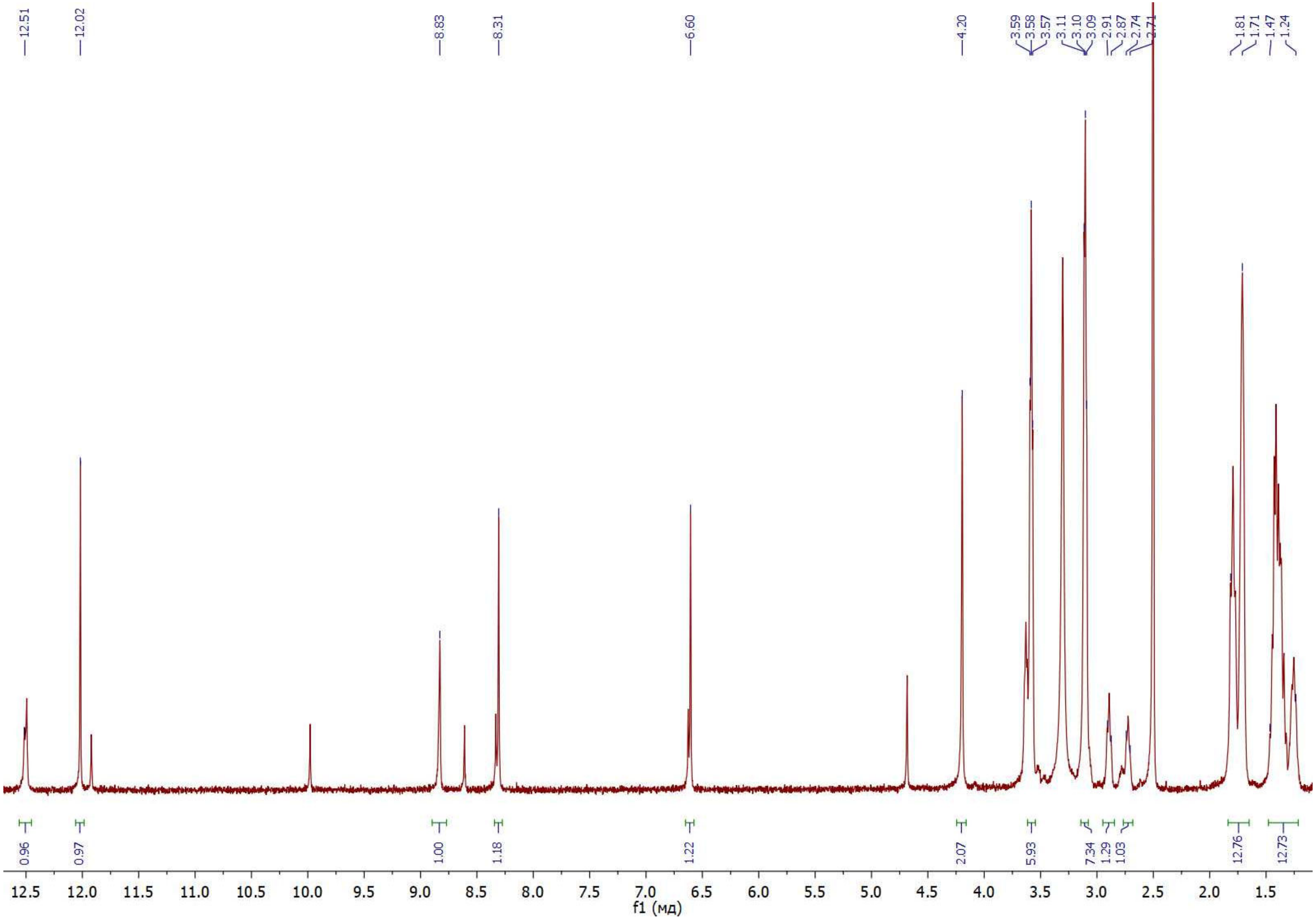

Fig. S28. ^{1}H NMR spectrum of compound **10c** (500 MHz, DMSO-d_6)

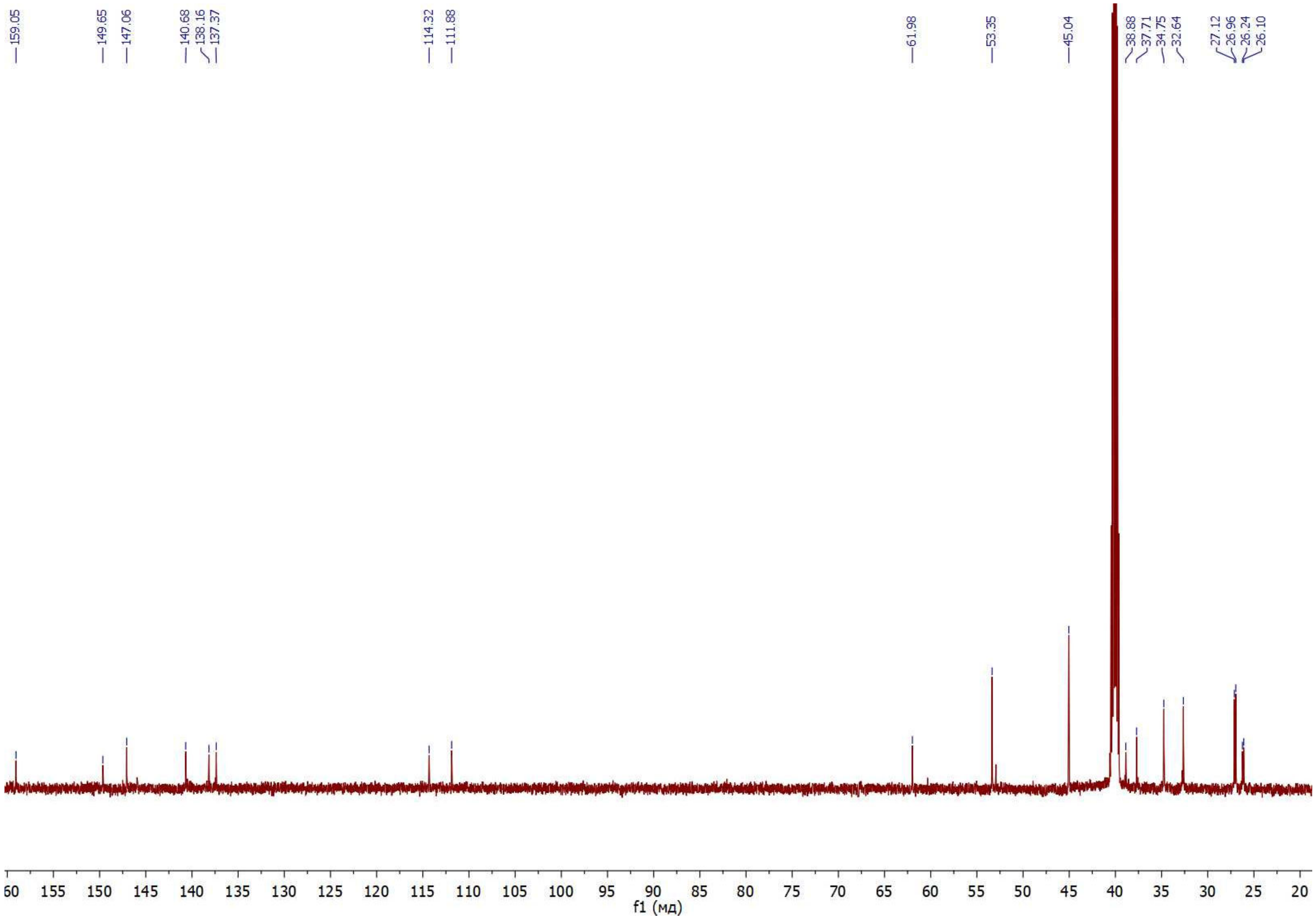

Fig. S29. ^{13}C-{^1H} NMR spectrum of compound **10c** (126 MHz, DMSO-d_6)

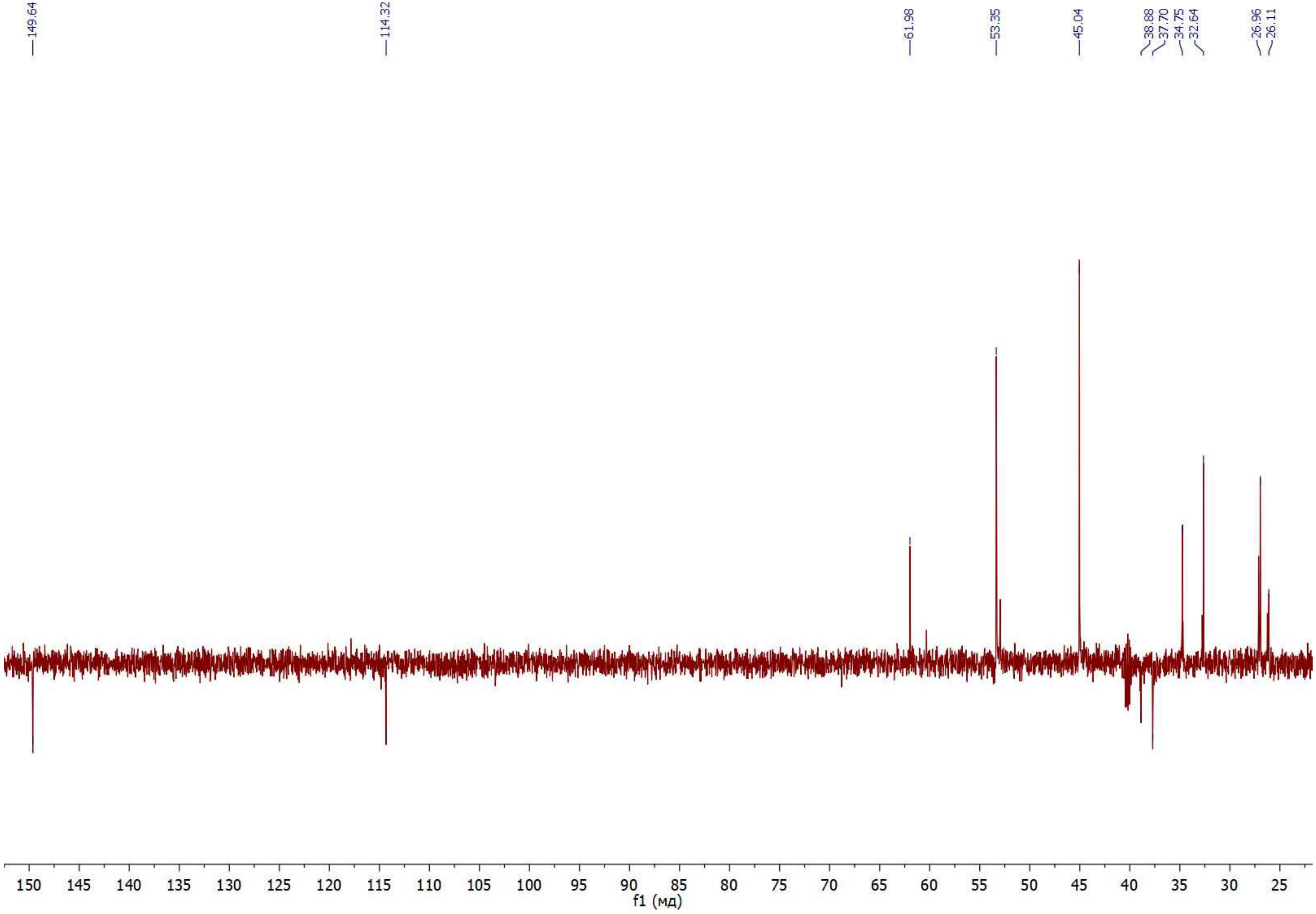

Fig. S30. ^{13}C (dept) NMR spectrum of compound **10c** (126 MHz, DMSO-d_6)

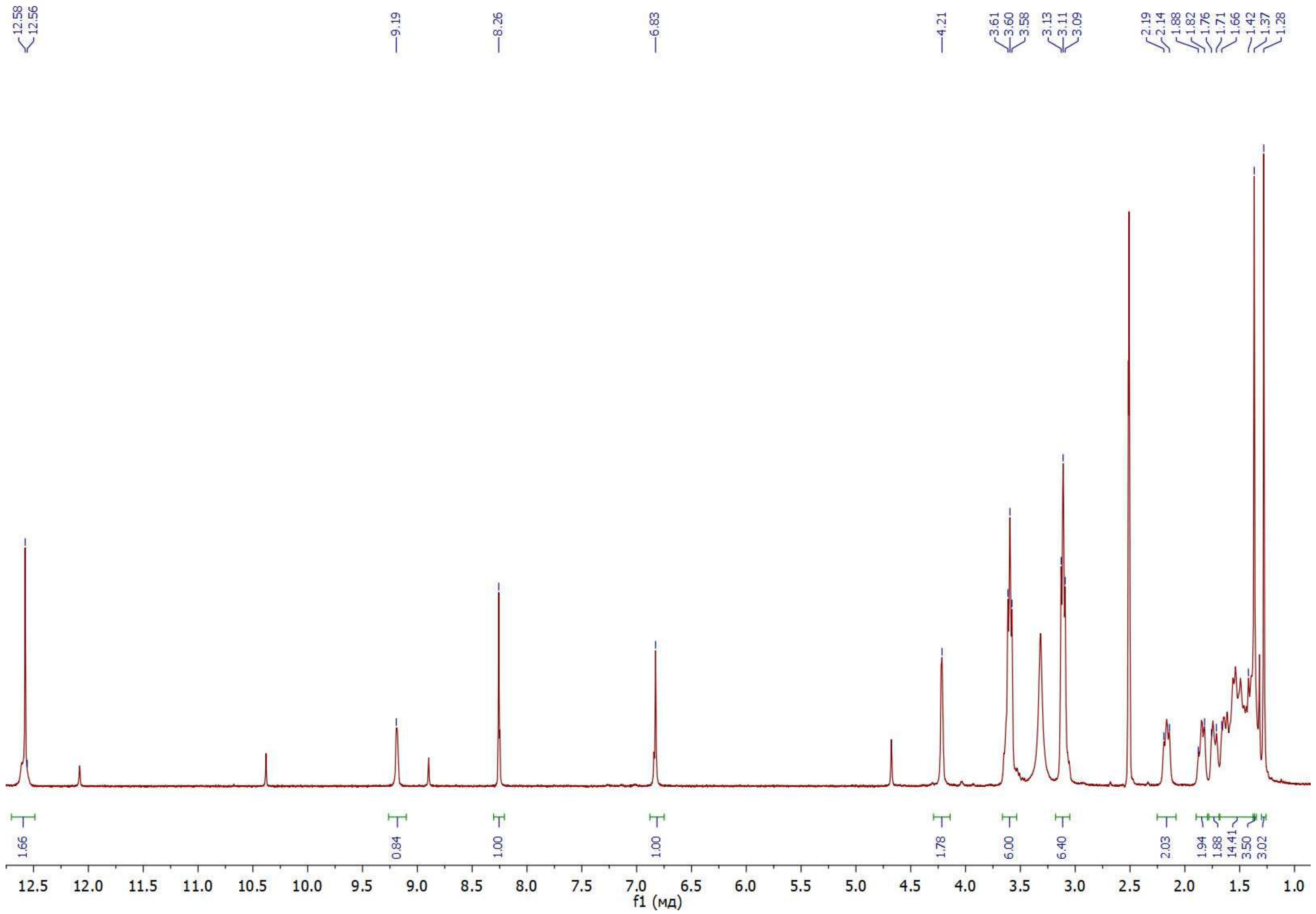

Fig. S31. ^{1}H NMR spectrum of compound **10d** (400 MHz, DMSO-d_6)

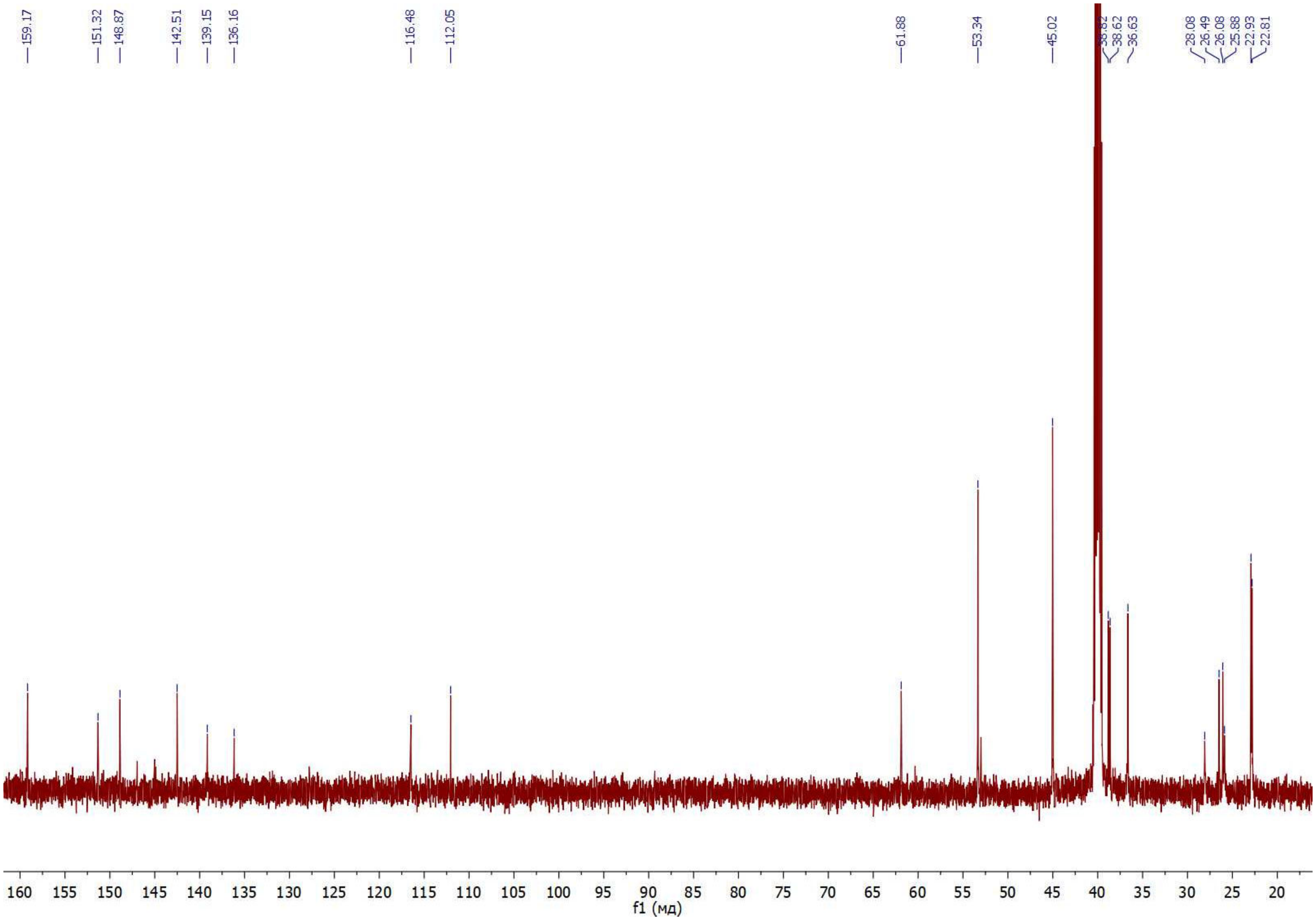

Fig. S32. ^{13}C-{^{1}H} NMR spectrum of compound **10d** (101 MHz, DMSO-d_6)

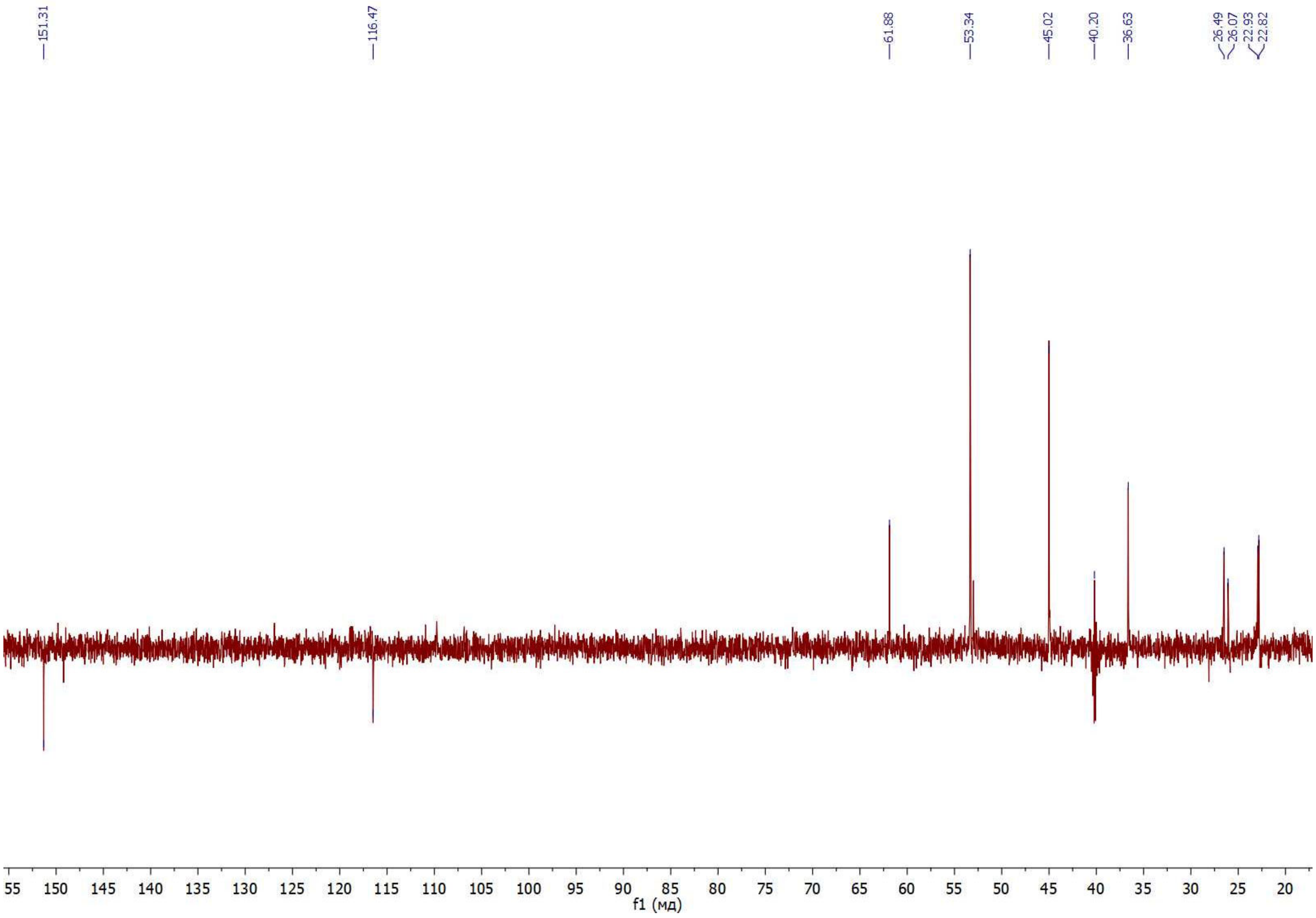

Fig. S33. ^{13}C (dept) NMR spectrum of compound **10d** (101 MHz, DMSO-d_6)

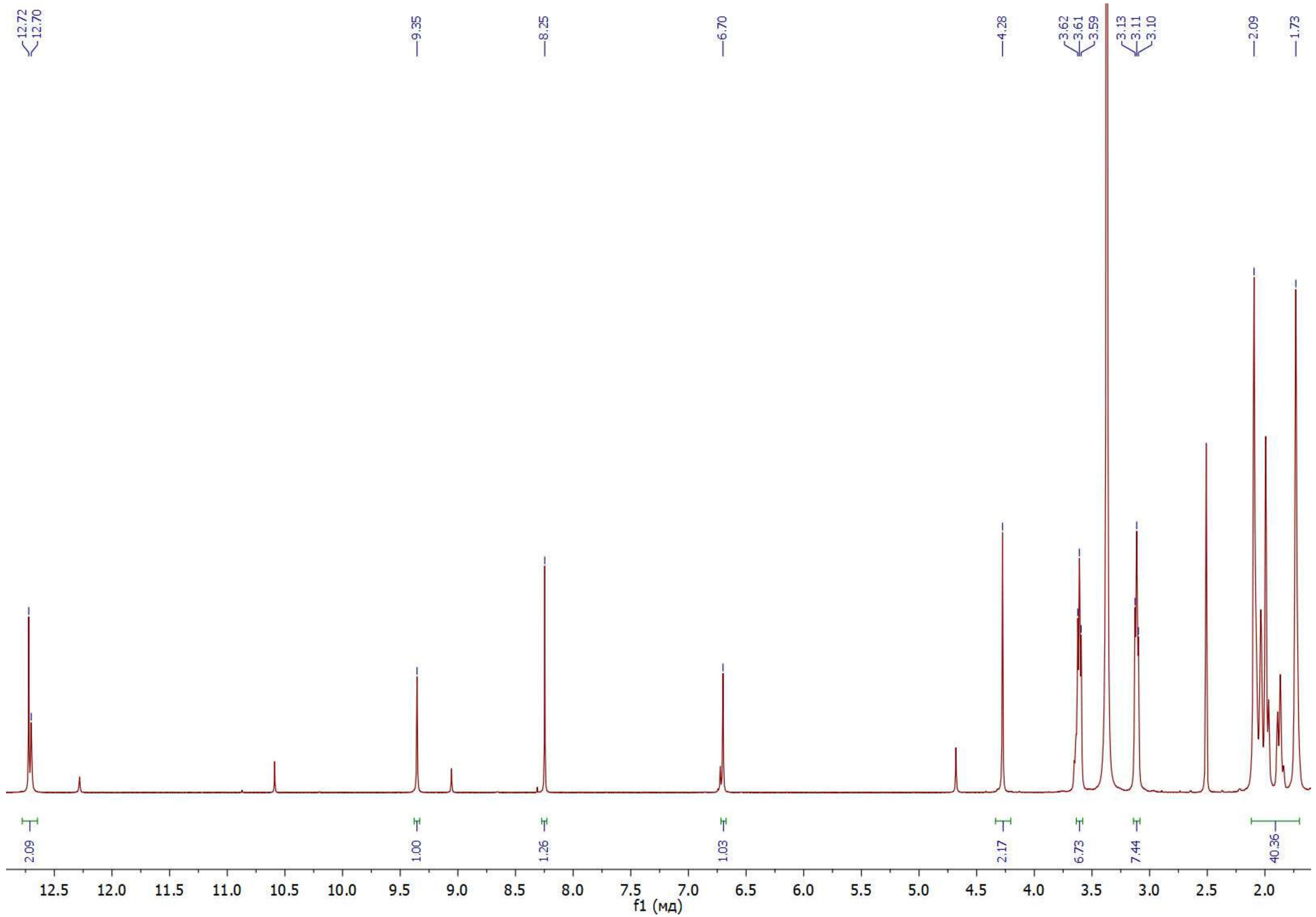

Fig. S34. ^{1}H NMR spectrum of compound **10e** (400 MHz, DMSO-d_6)

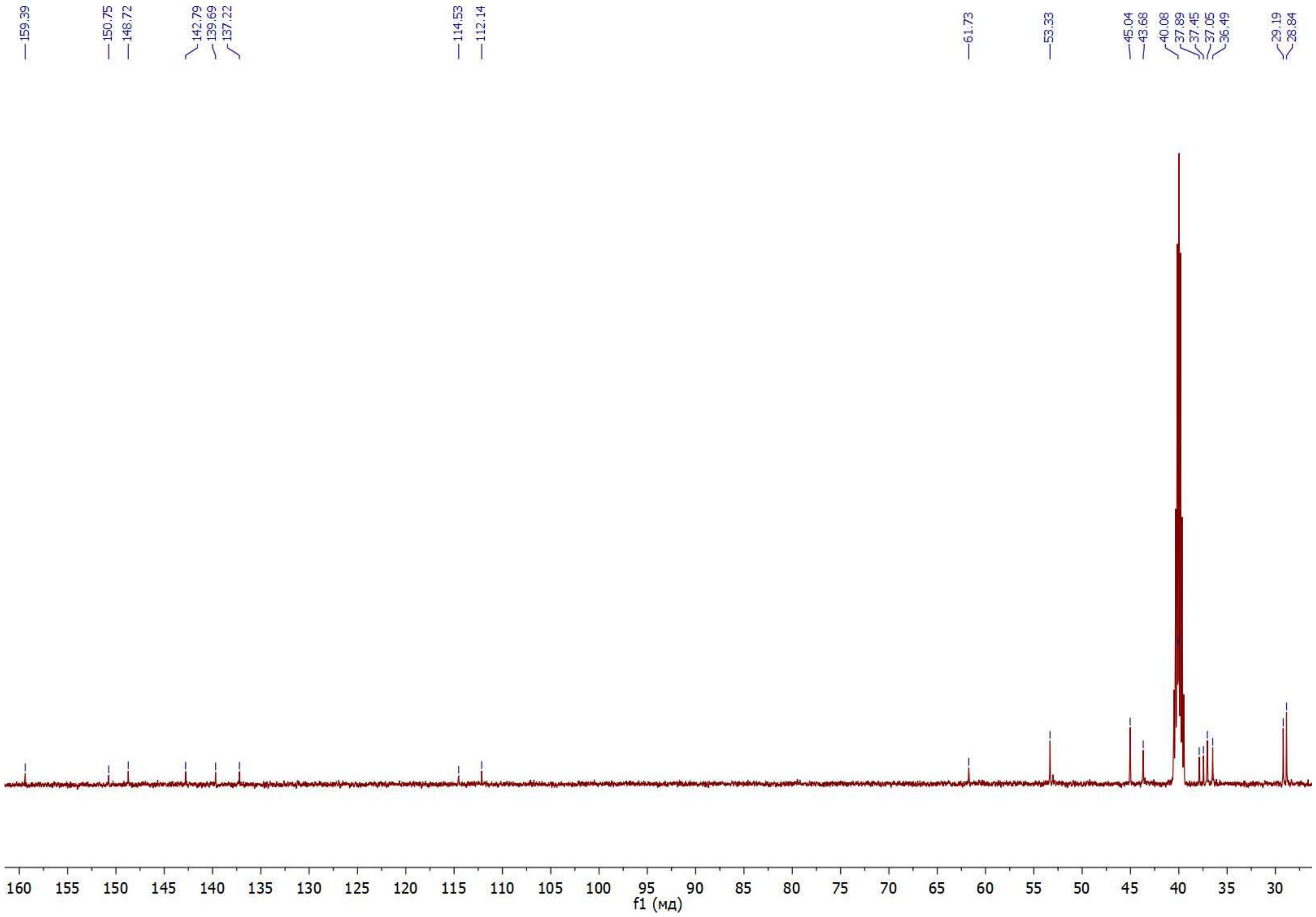

Fig. S35. ^{13}C-{^{1}H} NMR spectrum of compound **10e** (101 MHz, DMSO-d_6)

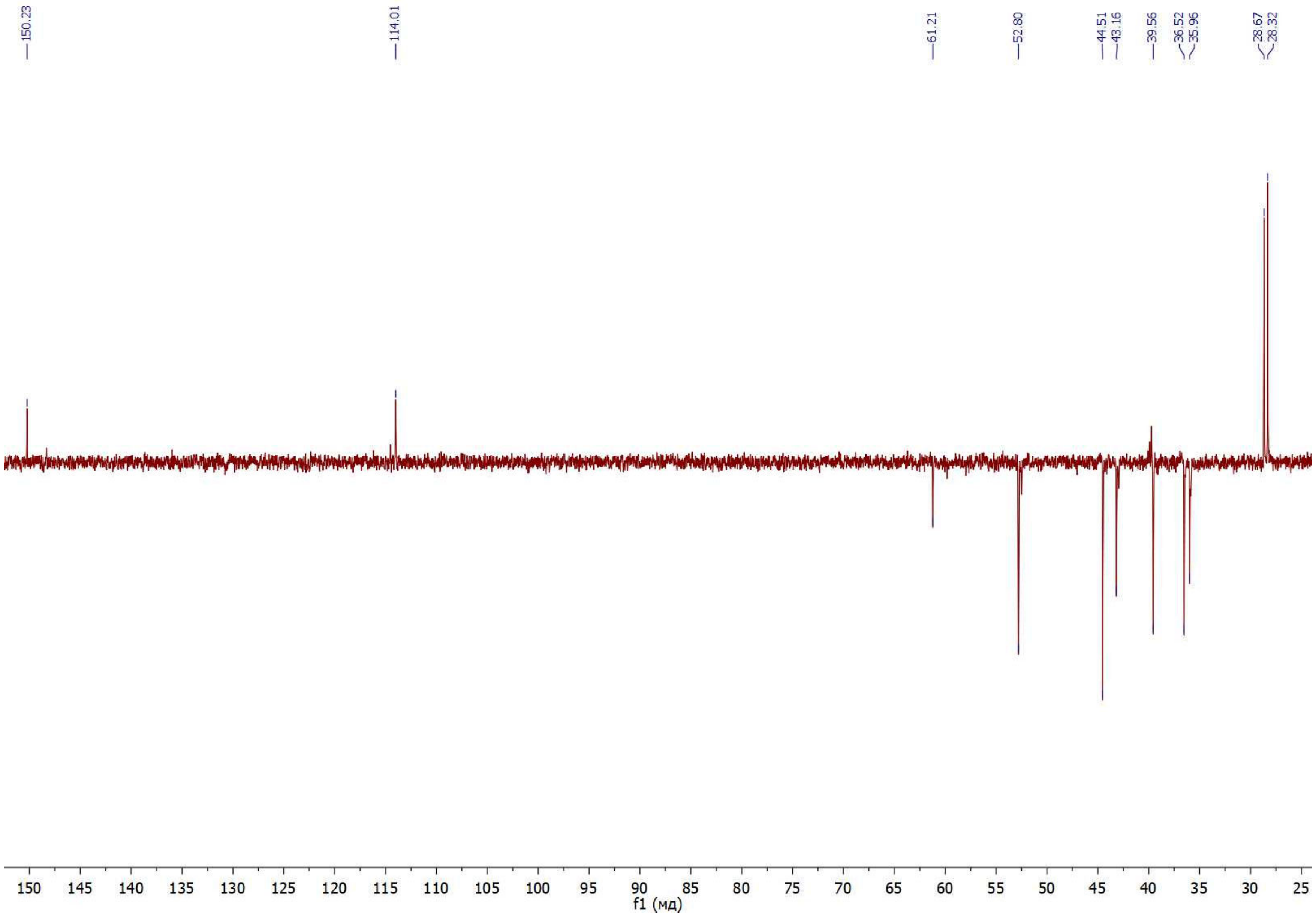

Fig. S36. ^{13}C (dept) NMR spectrum of compound **10e** (101 MHz, DMSO-d_6)

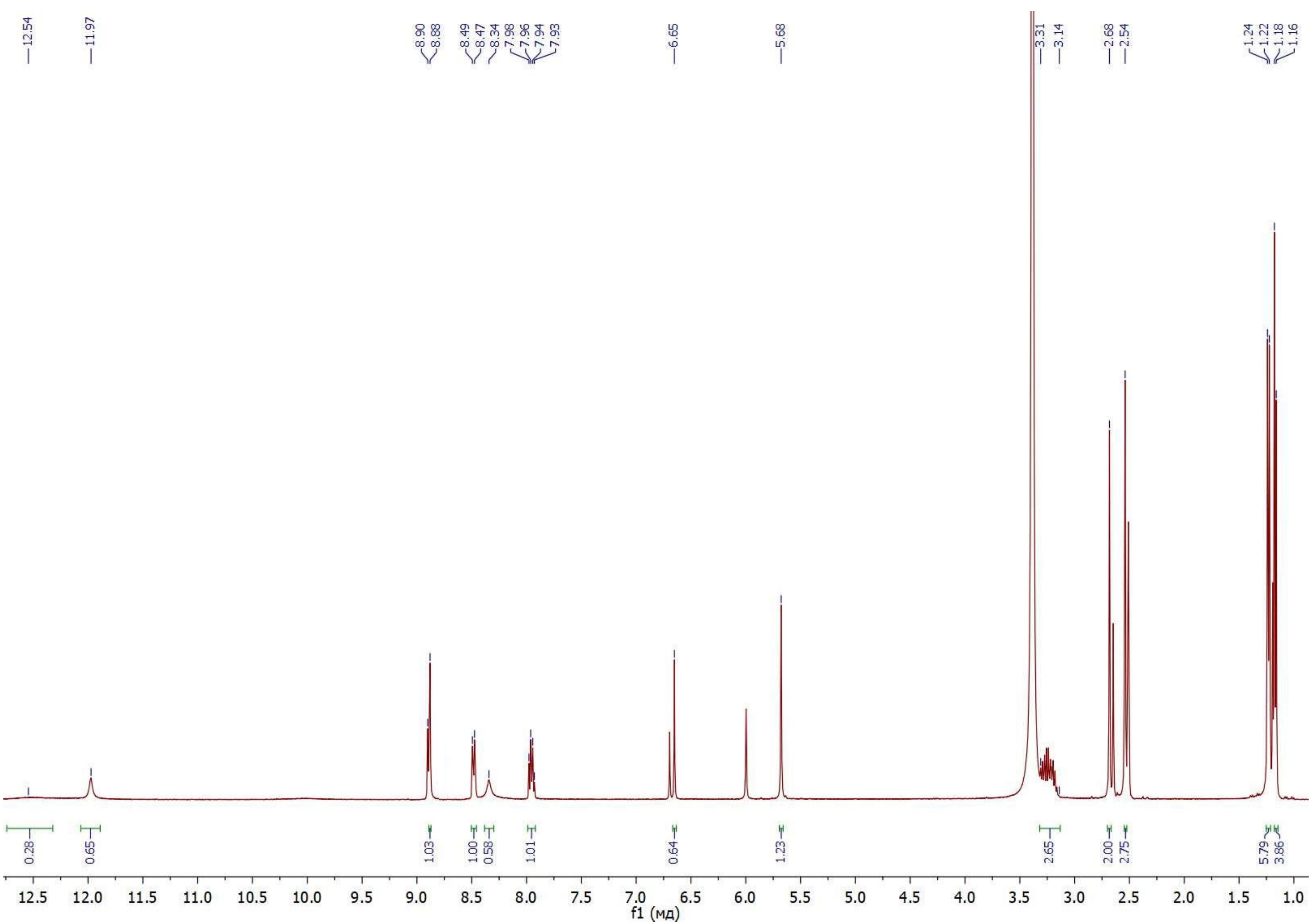

Fig. S37. ^{1}H NMR spectrum of compound **11a** (400 MHz, DMSO-d_6)

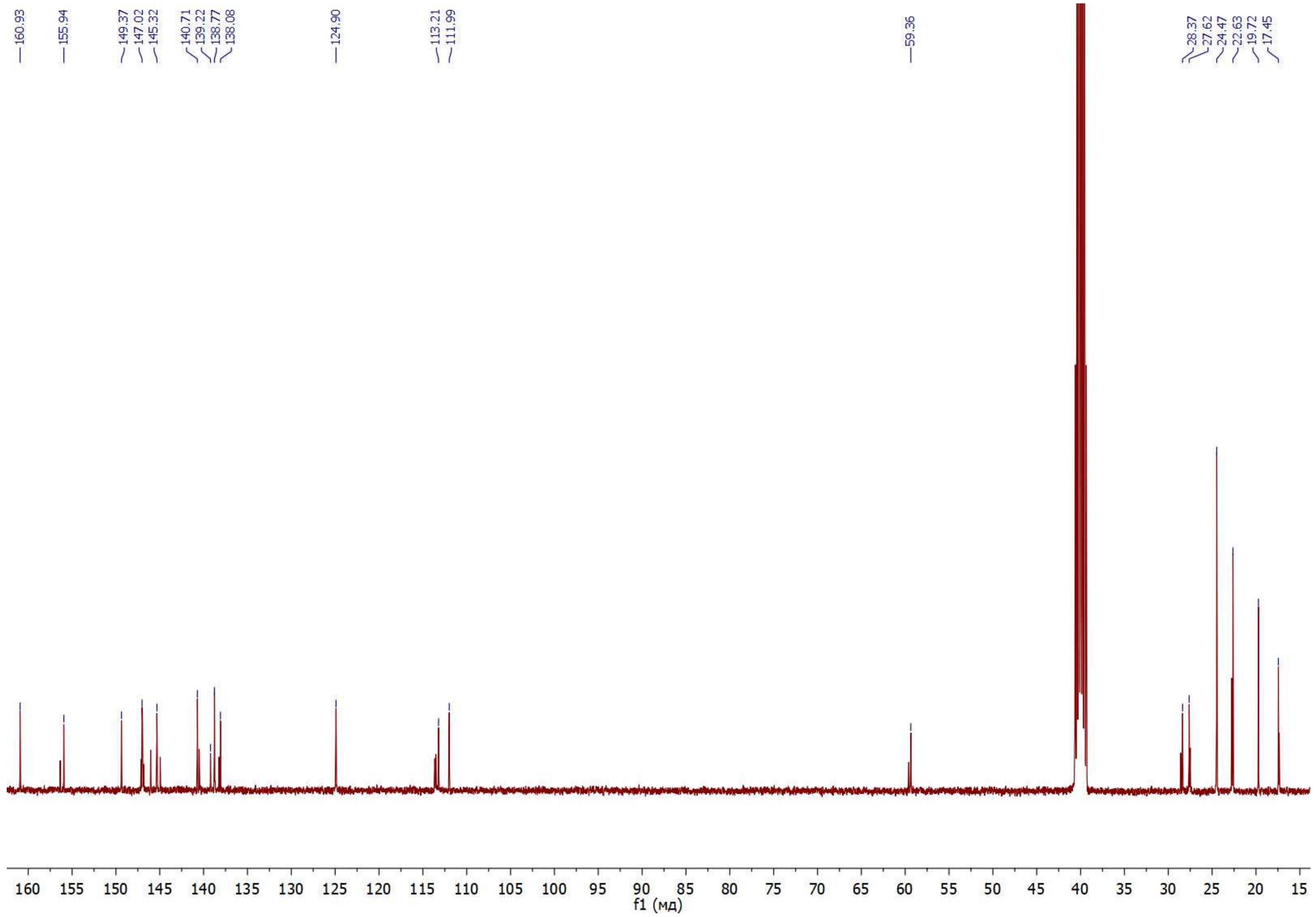

Fig. S38. ^{13}C-{^{1}H} NMR spectrum of compound **11a** (101 MHz, DMSO-d_6)

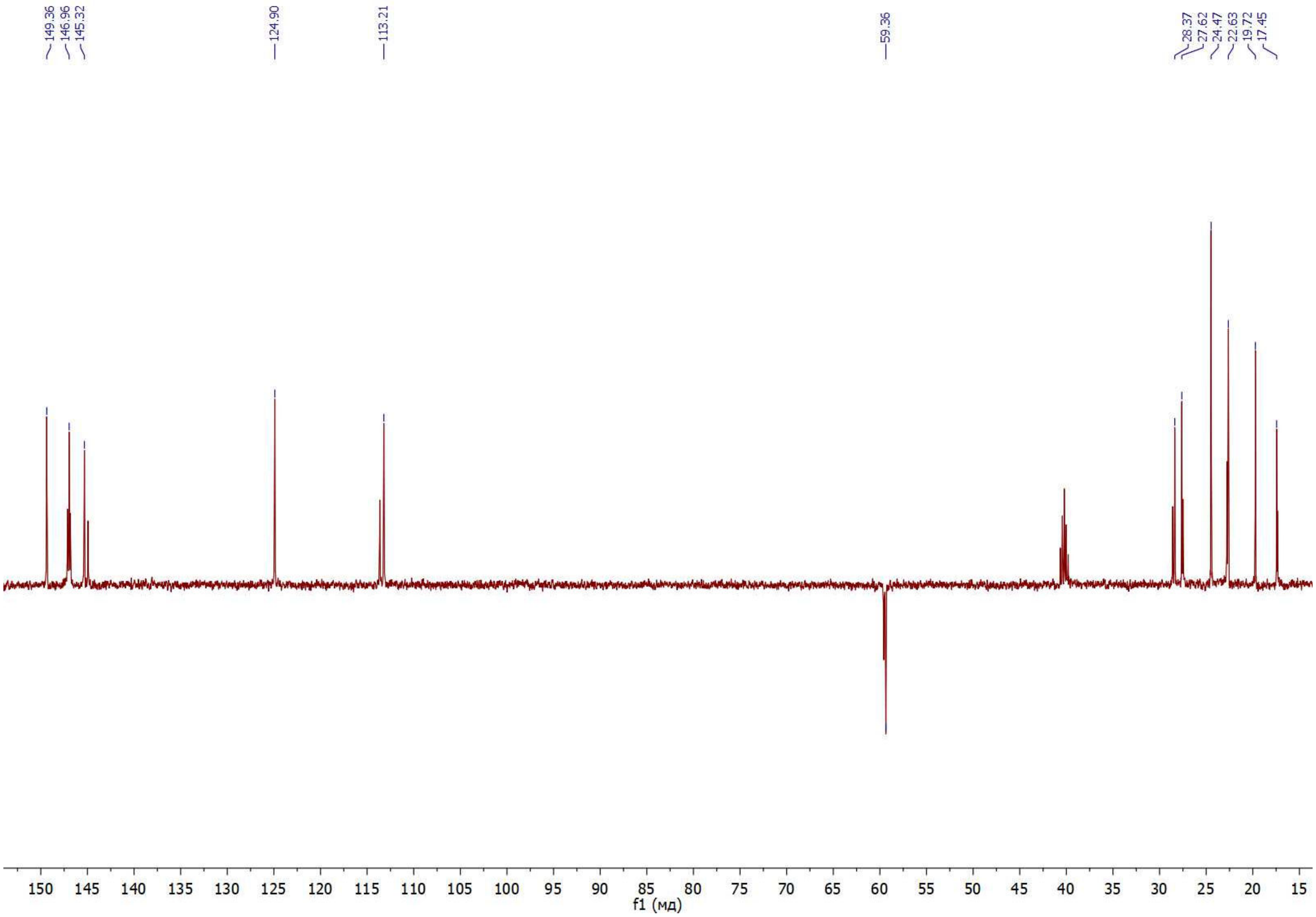

Fig. S39. ^{13}C (dept) NMR spectrum of compound **11a** (101 MHz, DMSO-d_6)

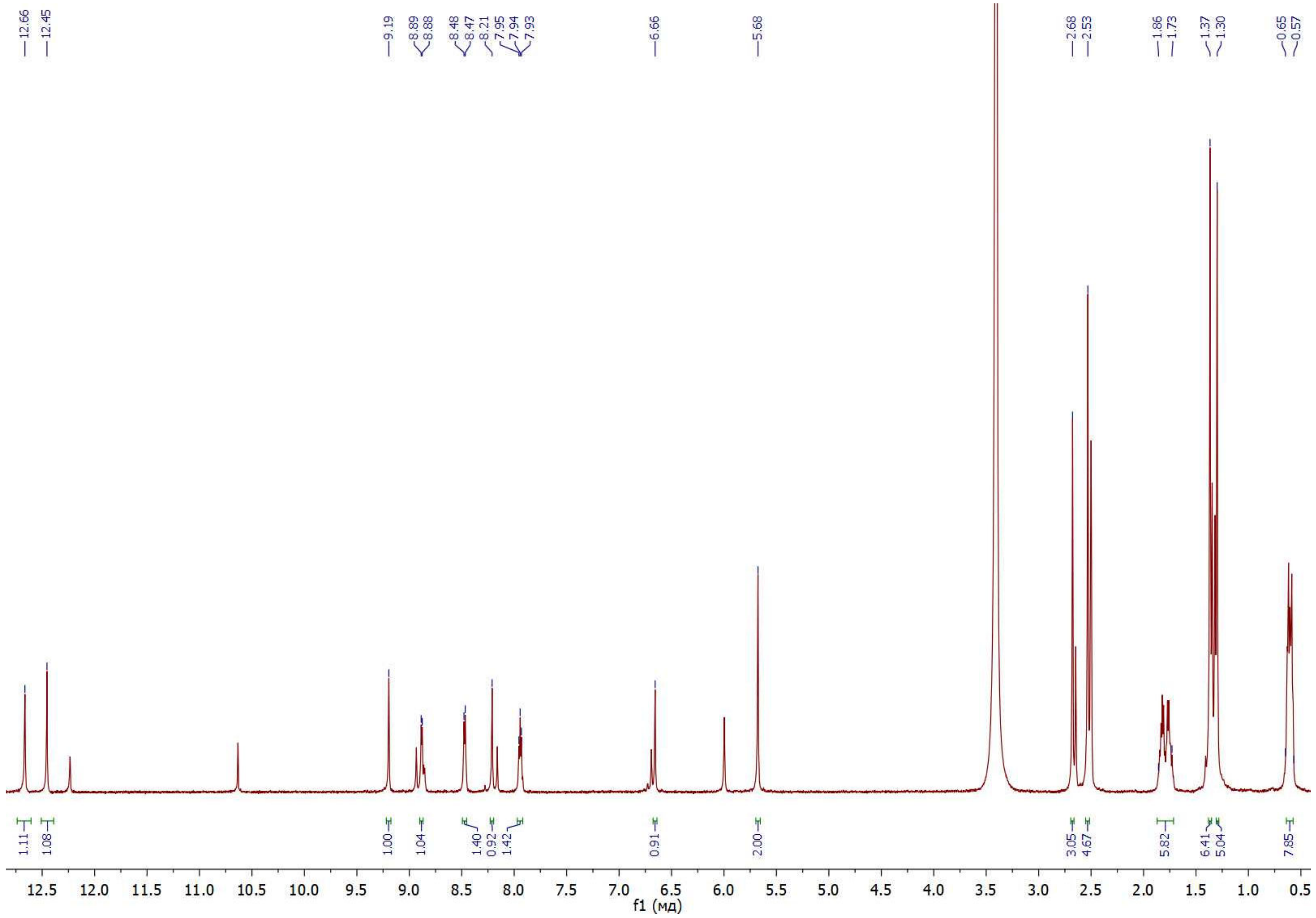

Fig. S40. ^{1}H NMR spectrum of compound **11b** (500 MHz, DMSO-d_6)

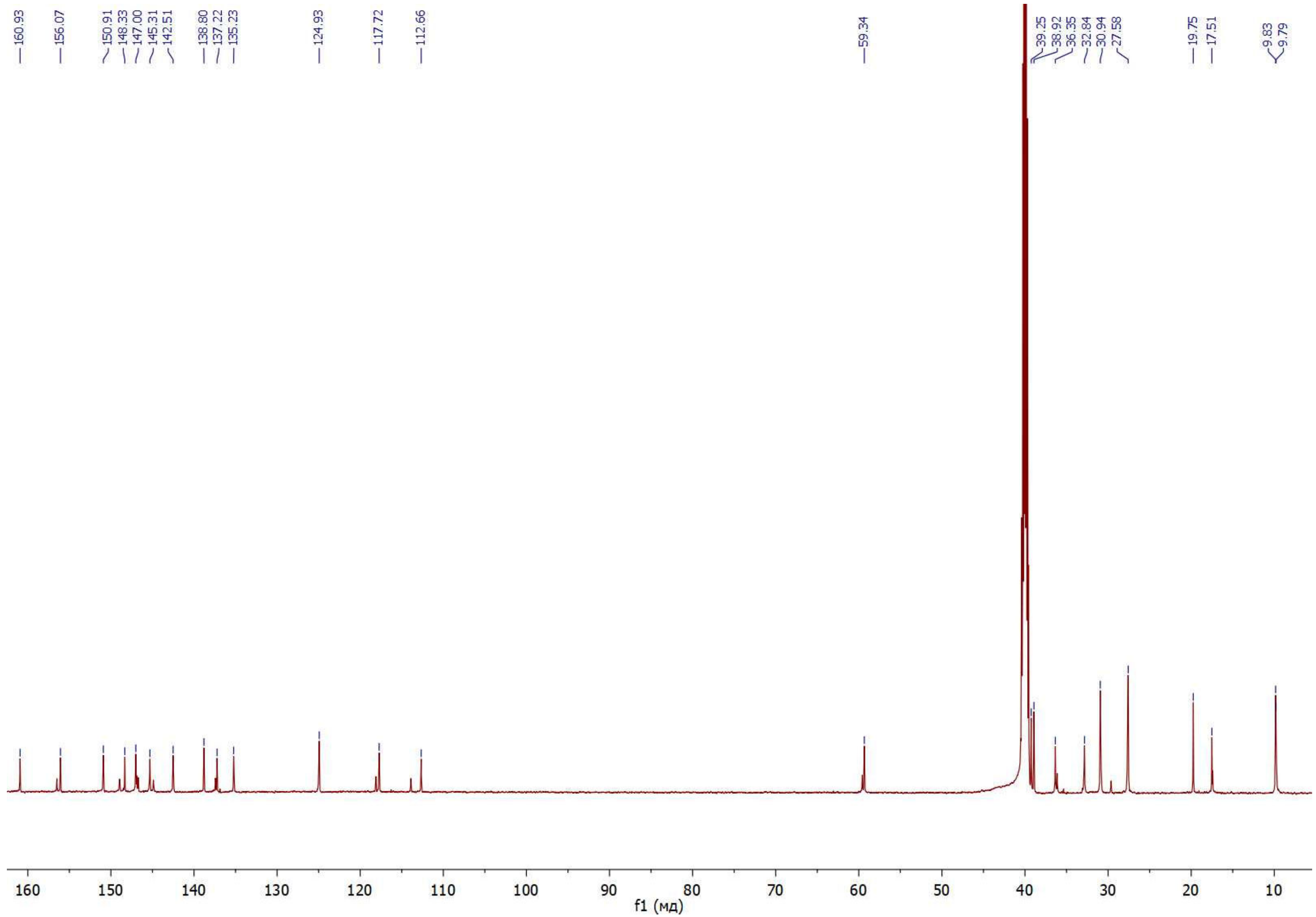

Fig. S41. ^{13}C-{^{1}H} NMR spectrum of compound **11b** (126 MHz, DMSO-d_6)

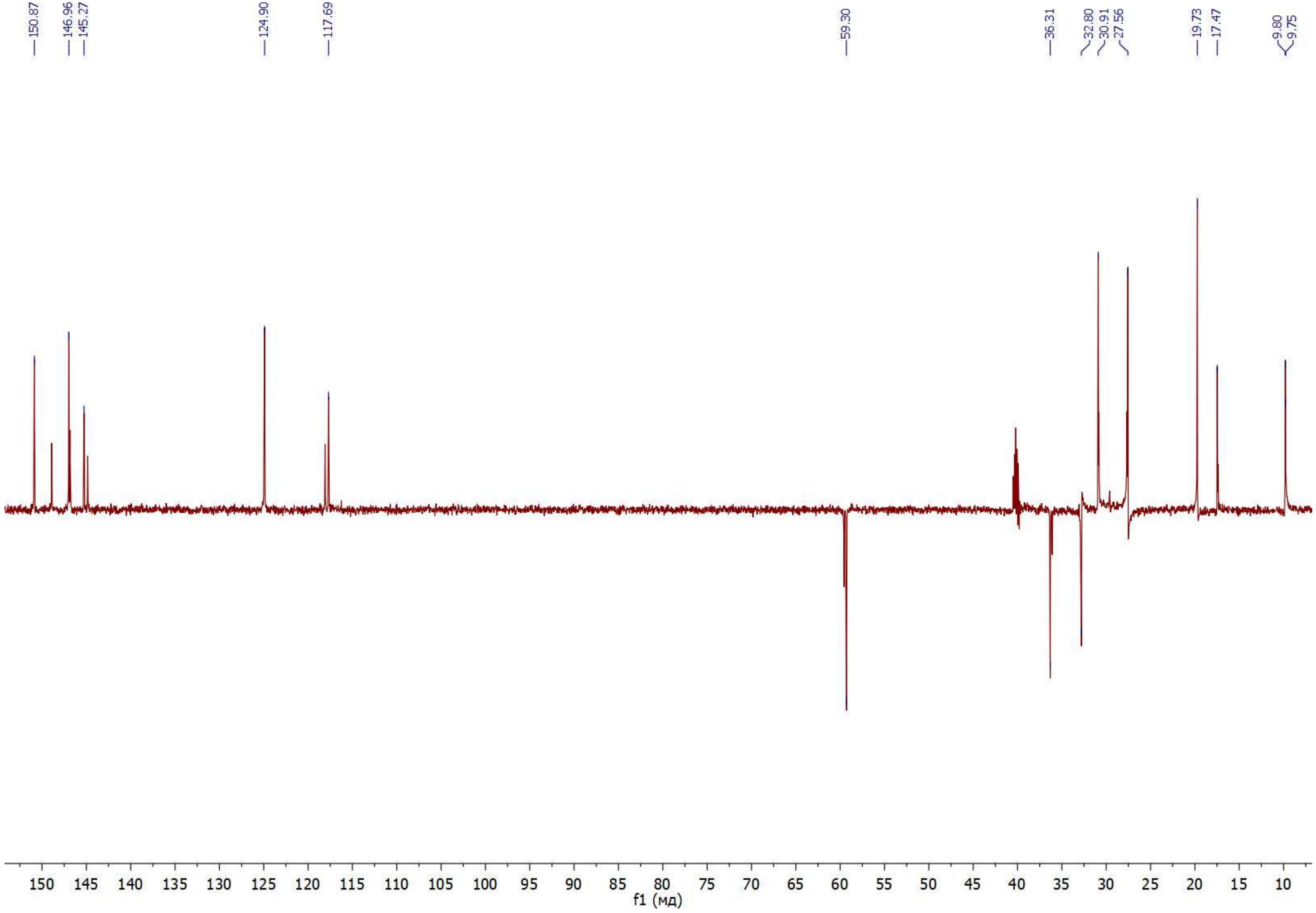

Fig. S42. ^{13}C (dept) NMR spectrum of compound **11b** (126 MHz, DMSO-d_6)

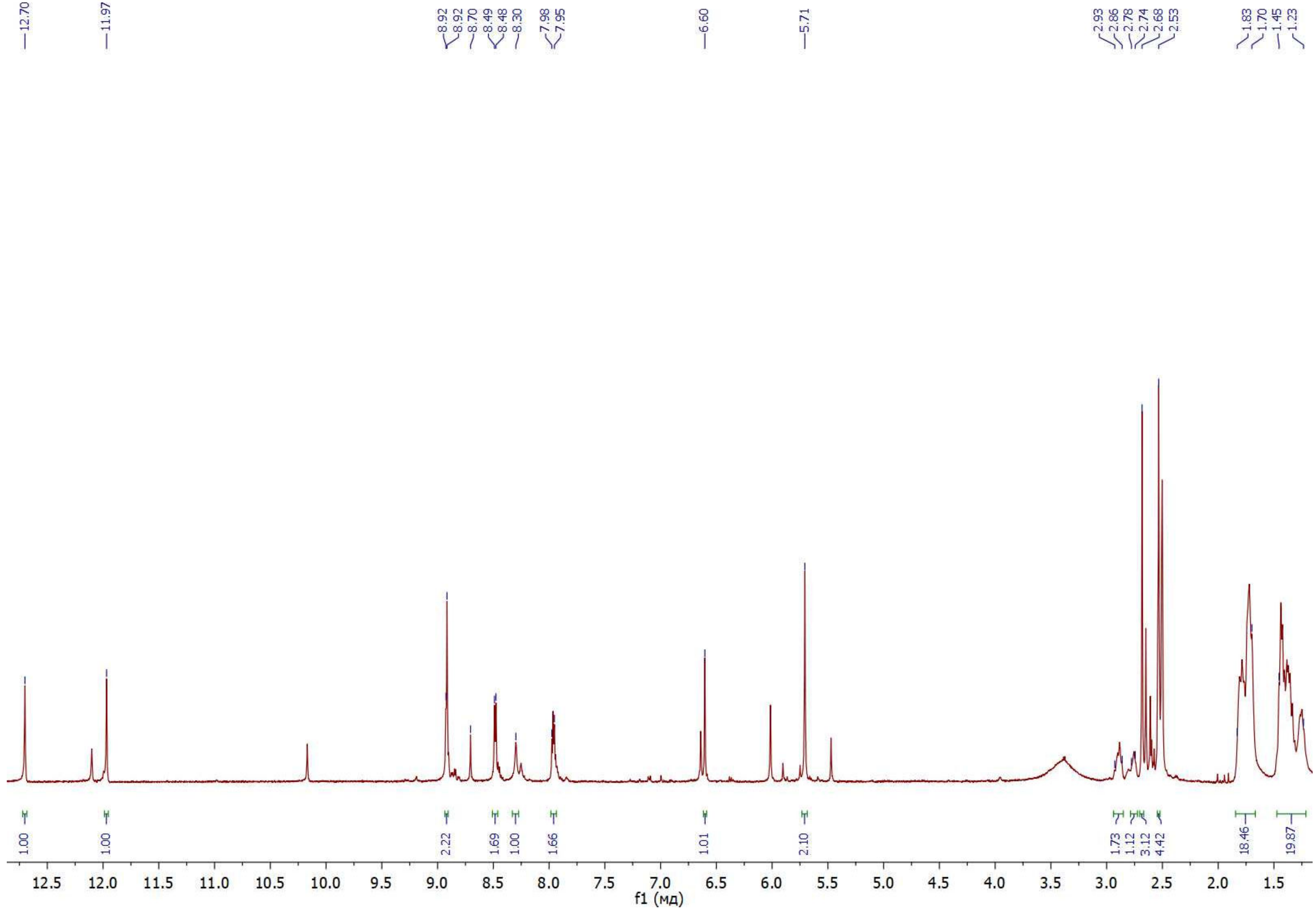

Fig. S43. ^{1}H NMR spectrum of compound **11c** (400 MHz, DMSO-d_6)

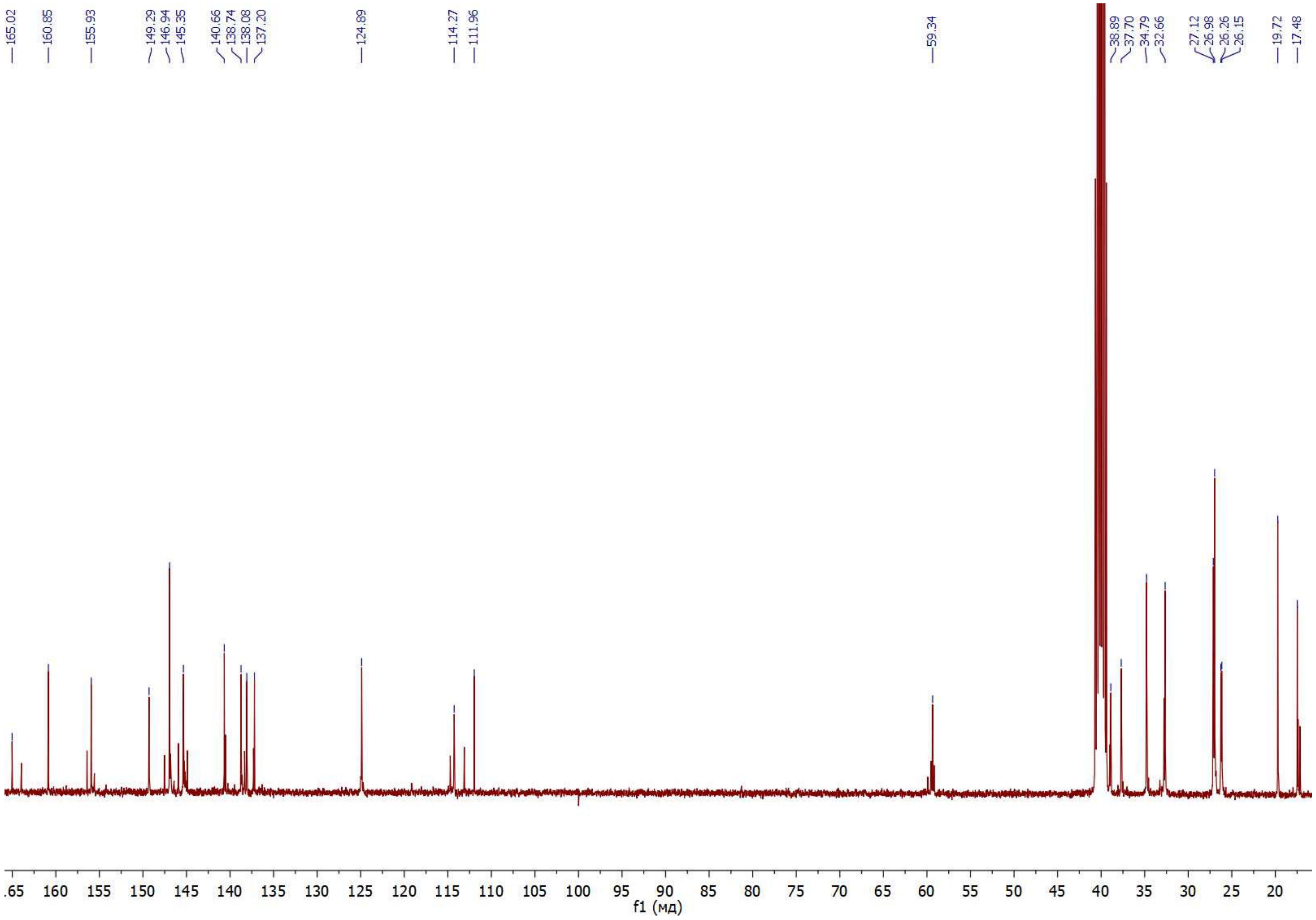

Fig. S44. ^{13}C-{^{1}H} NMR spectrum of compound **11c** (101 MHz, DMSO-d_6)

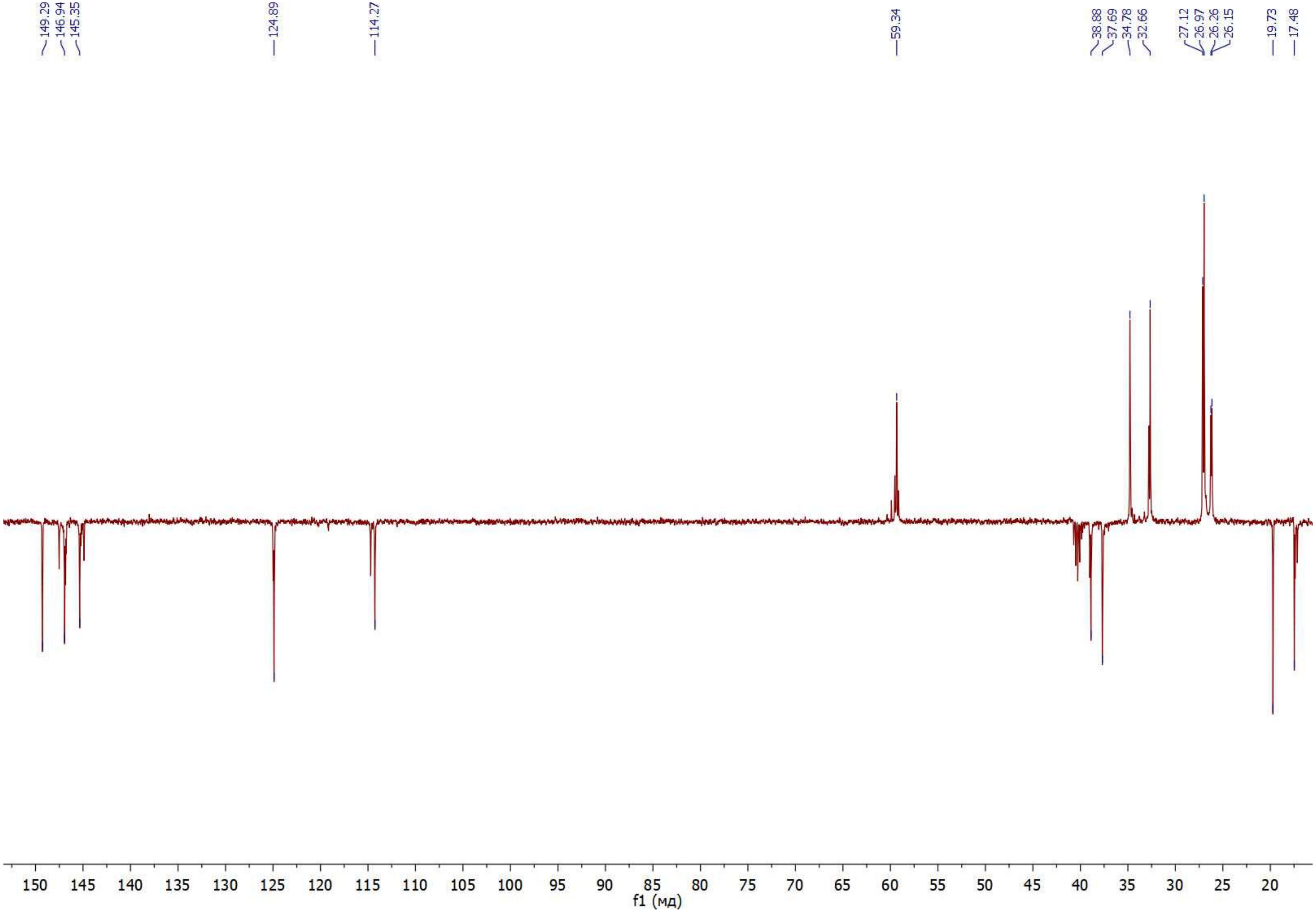

Fig. S45. ^{13}C (dept) NMR spectrum of compound **11c** (101 MHz, DMSO-d_6)

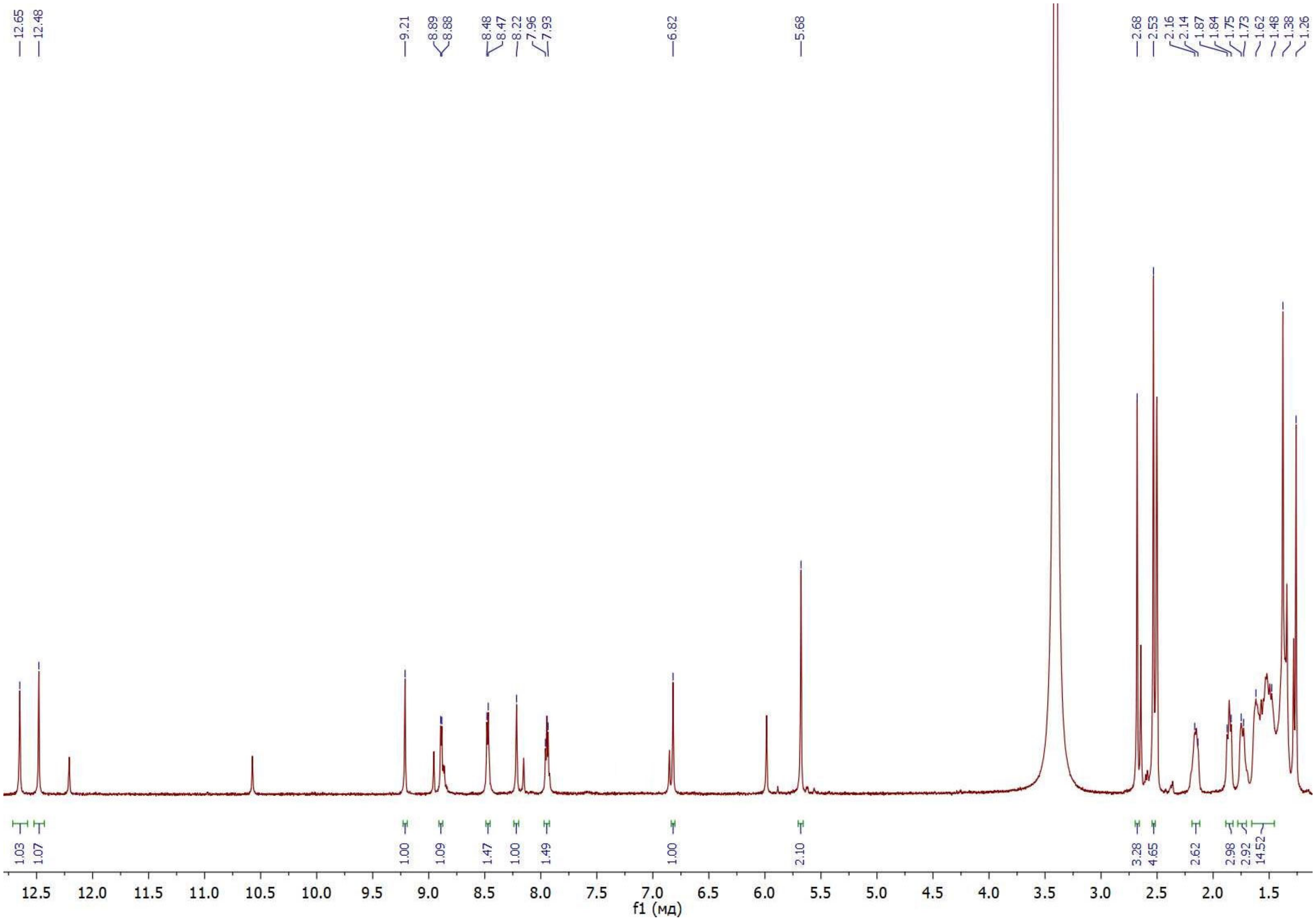

Fig. S46. ^{1}H NMR spectrum of compound **11d** (600 MHz, DMSO-d_6)

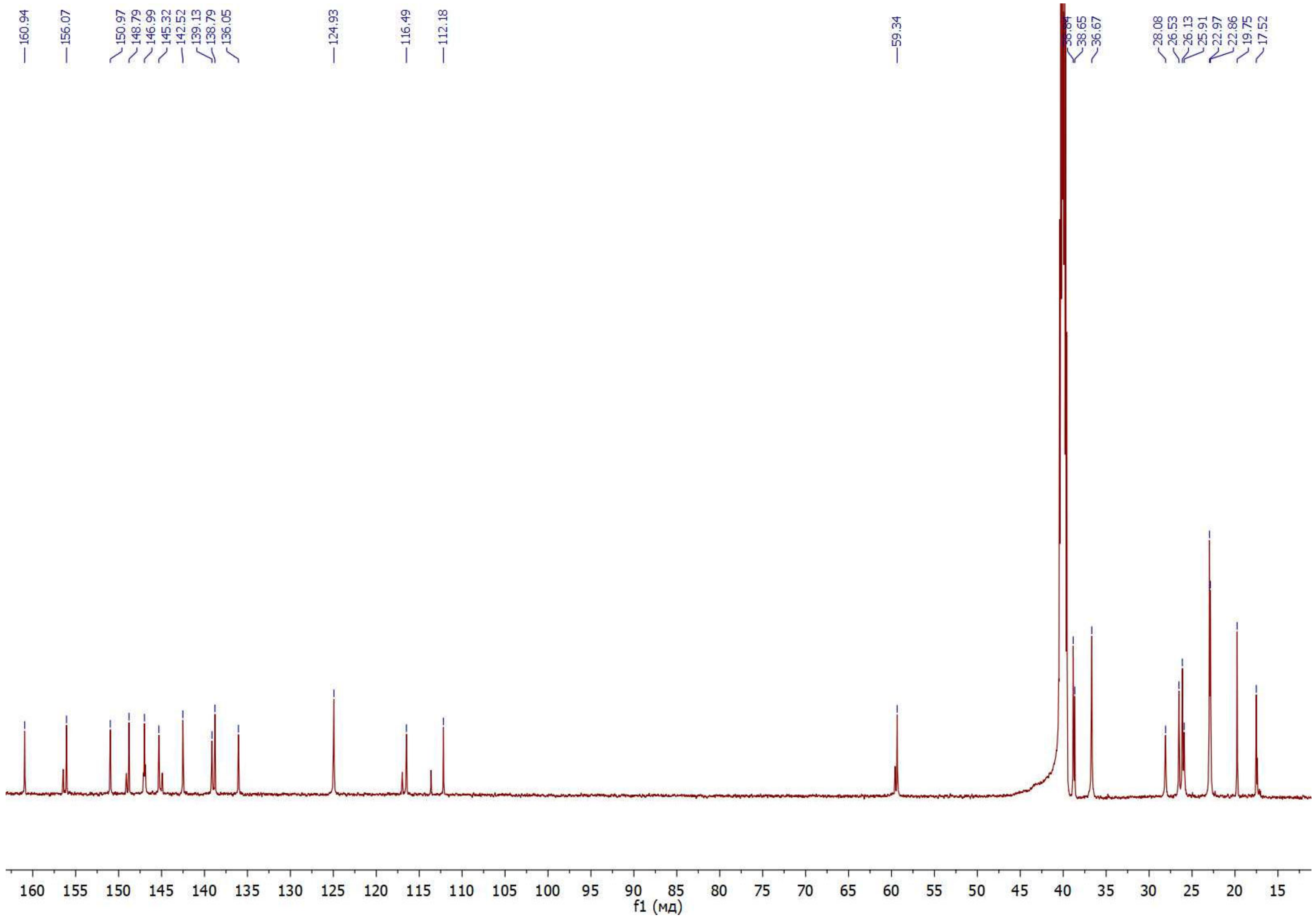

Fig. S47. ^{13}C-{^{1}H} NMR spectrum of compound **11d** (151 MHz, DMSO-d_6)

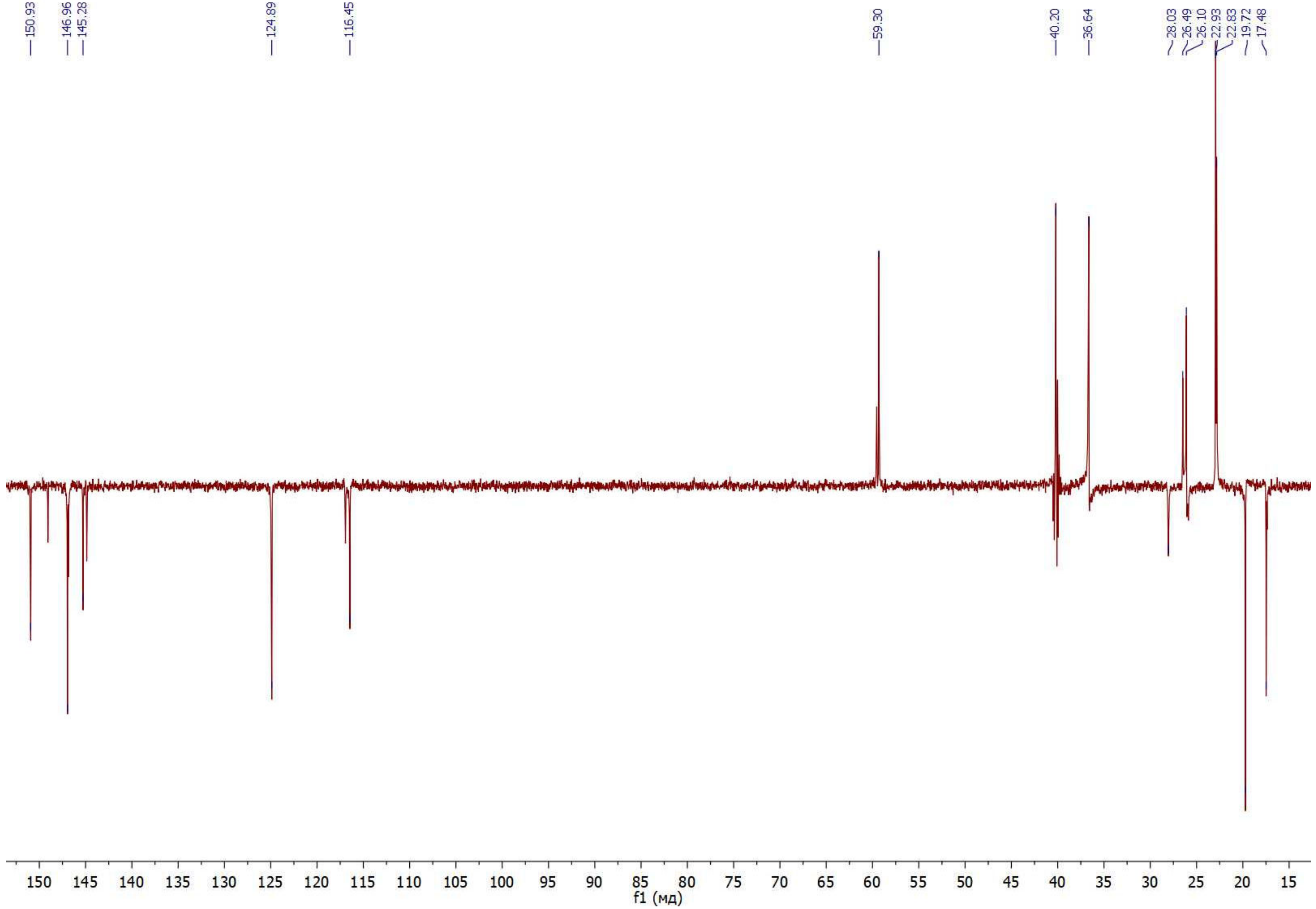

Fig. S48. ^{13}C (dept) NMR spectrum of compound **11d** (151 MHz, DMSO-d_6)

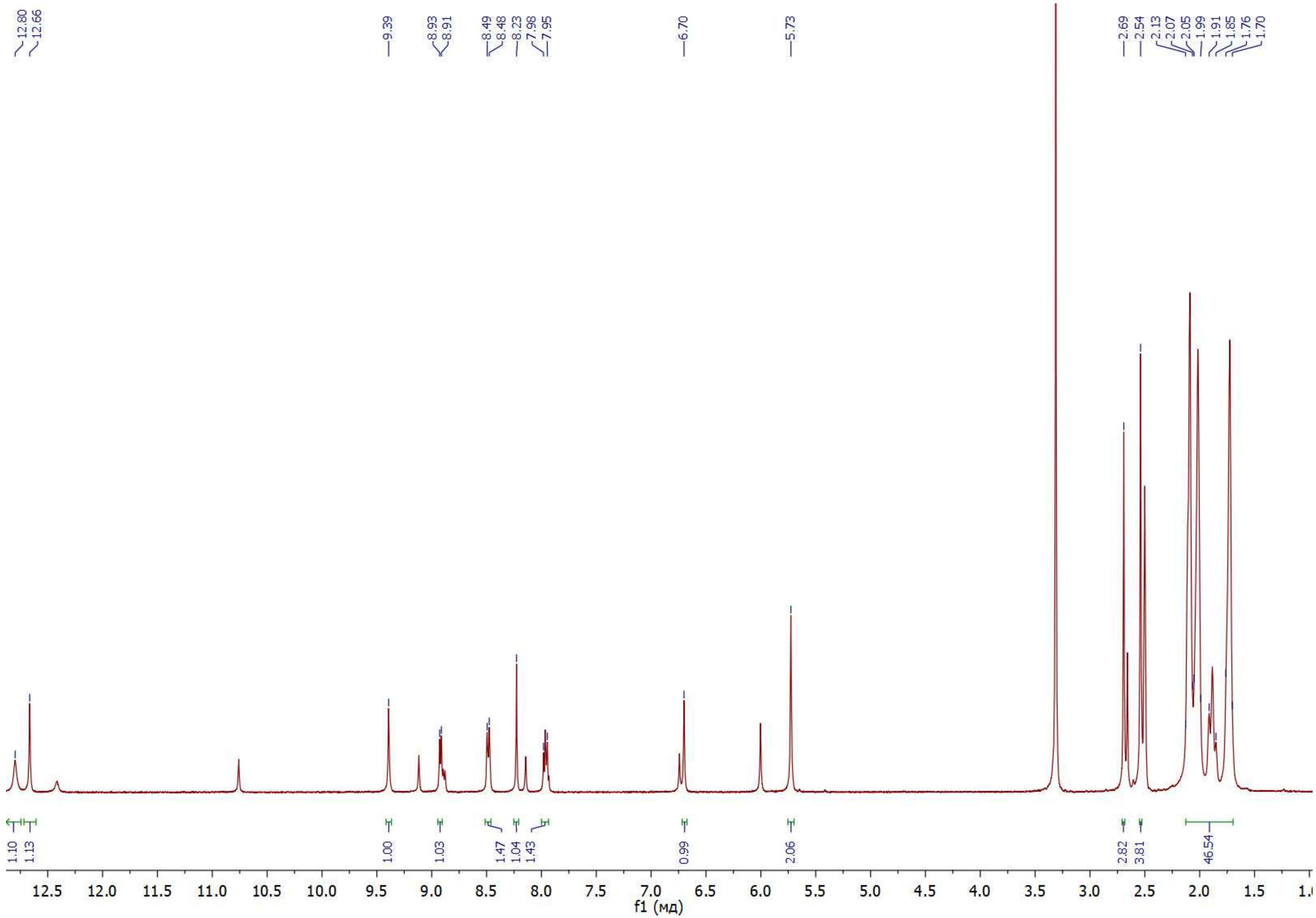

Fig. S49. ^{1}H NMR spectrum of compound **11e** (400 MHz, DMSO-d_6)

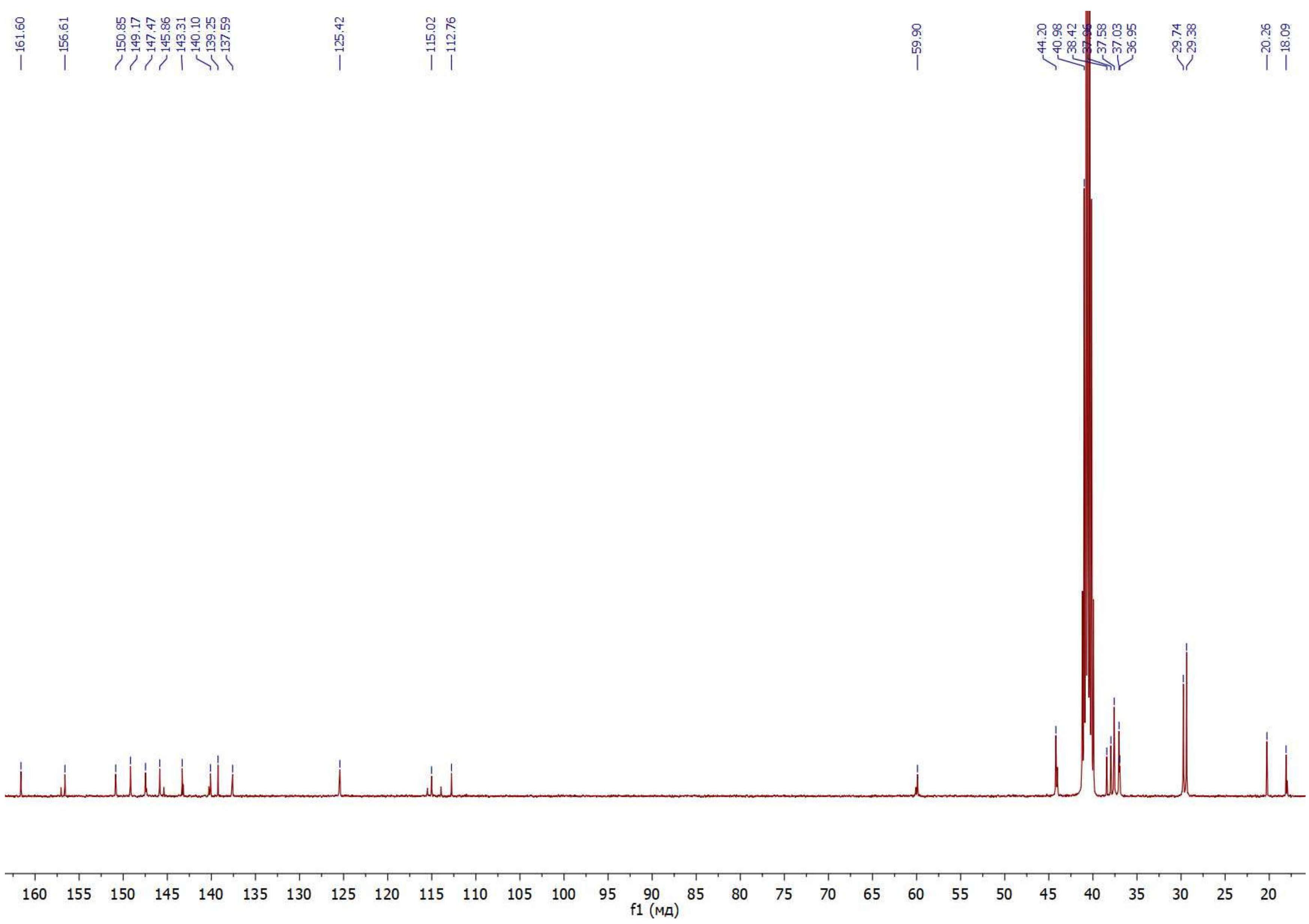

Fig. S50. ^{13}C-{^{1}H} NMR spectrum of compound **11e** (101 MHz, DMSO-d_6)

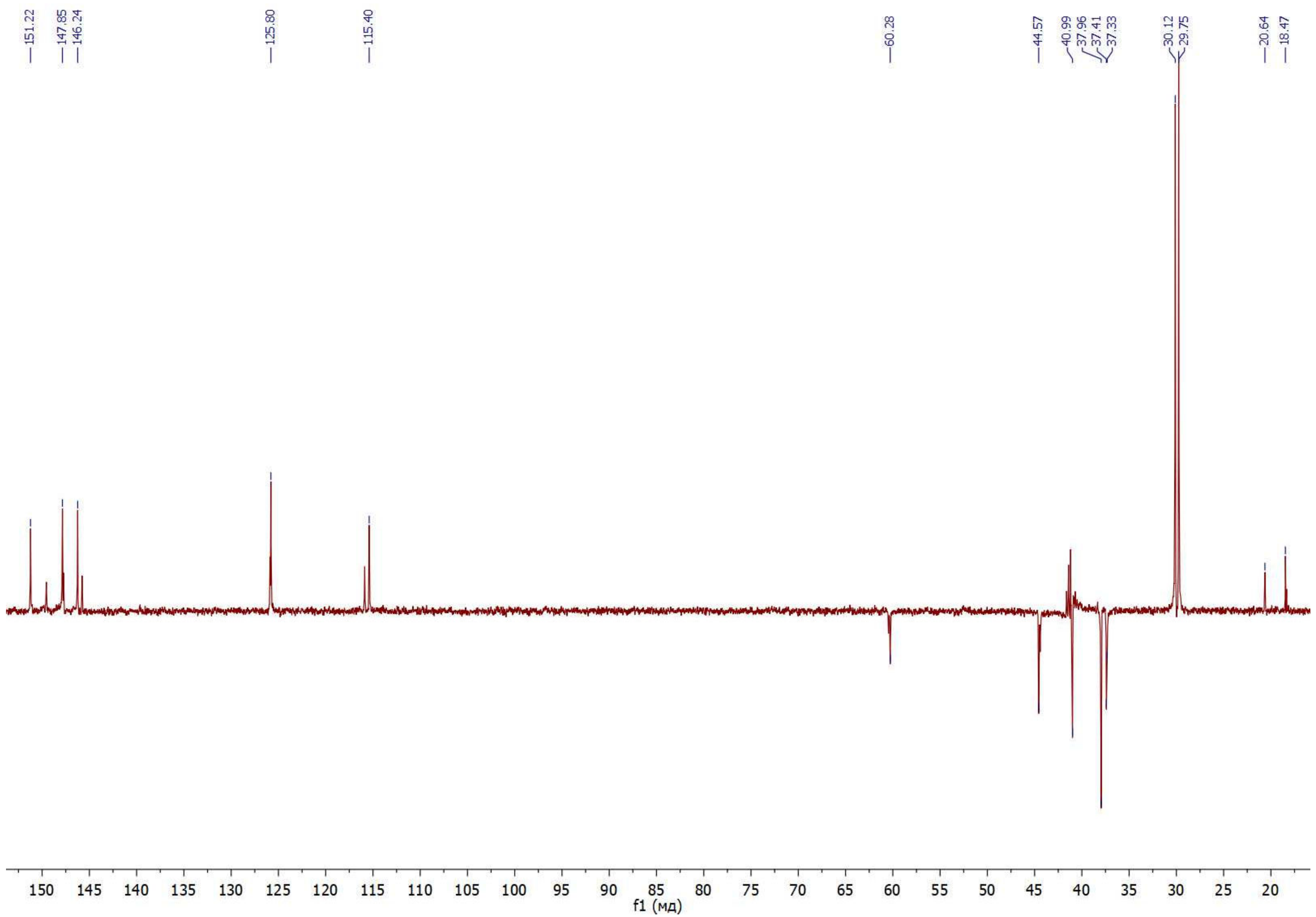

Fig. S51. ^{13}C (dept) NMR spectrum of compound **11e** (101 MHz, DMSO-d_6)
